# Metabolome Analysis Reveals Betaine Lipids as Major Source for Triglyceride Formation, and the Accumulation of Sedoheptulose during Nitrogen-Starvation of *Phaeodactylum tricornutum*

**DOI:** 10.1371/journal.pone.0164673

**Published:** 2016-10-13

**Authors:** Jennifer Popko, Cornelia Herrfurth, Kirstin Feussner, Till Ischebeck, Tim Iven, Richard Haslam, Mary Hamilton, Olga Sayanova, Jonathan Napier, Inna Khozin-Goldberg, Ivo Feussner

**Affiliations:** 1 Georg-August-University, Albrecht-von-Haller-Institute for Plant Sciences, Department of Plant Biochemistry, Justus-von-Liebig-Weg 11, 37077, Göttingen, Germany; 2 Rothamsted Research, Biological Chemistry, Harpenden, AL5 2JQ, United Kingdom; 3 Microalgal Biotechnology Laboratory, The French Associates Institute for Agriculture and Biotechnology of Drylands, The Jacob Blaustein Institutes for Desert Research, Ben-Gurion University of the Negev, Sede Boqer Campus 8499000, Midreshet Ben-Gurion, Israel; 4 Georg-August-University, Goettingen Center for Molecular Biosciences (GZMB), Department of Plant Biochemistry, Justus-von-Liebig-Weg 11, 37077, Göttingen, Germany; 5 Georg-August-University, International Center for Advanced Studies of Energy Conversion (ICASEC), Department of Plant Biochemistry, Justus-von-Liebig-Weg 11, 37077, Göttingen, Germany; University of Hyderabad, INDIA

## Abstract

Oleaginous microalgae are considered as a promising resource for the production of biofuels. Especially diatoms arouse interest as biofuel producers since they are most productive in carbon fixation and very flexible to environmental changes in the nature. Naturally, triacylglycerol (TAG) accumulation in algae only occurs under stress conditions like nitrogen-limitation. We focused on *Phaeodactylum* strain Pt4 (UTEX 646), because of its ability to grow in medium with low salinity and therefore being suited when saline water is less available or for wastewater cultivation strategies. Our data show an increase in neutral lipids during nitrogen-depletion and predominantly 16:0 and 16:1(n-7) accumulated in the TAG fraction. The molecular species composition of TAG suggests a remodeling primarily from the betaine lipid diacylglyceroltrimethylhomoserine (DGTS), but a contribution of the chloroplast galactolipid monogalactosyldiacylglycerol (MGDG) cannot be excluded. Interestingly, the acyl-CoA pool is rich in 20:5(n-3) and 22:6(n-3) in all analyzed conditions, but these fatty acids are almost excluded from TAG. Other metabolites most obviously depleted under nitrogen-starvation were amino acids, lyso-phospholipids and tricarboxylic acid (TCA) cycle intermediates, whereas sulfur-containing metabolites as dimethylsulfoniopropionate, dimethylsulfoniobutyrate and methylsulfate as well as short acyl chain carnitines, propanoyl-carnitine and butanoyl-carnitine increased upon nitrogen-starvation. Moreover, the Calvin cycle may be de-regulated since sedoheptulose accumulated after nitrogen-depletion. Together the data provide now the basis for new strategies to improve lipid production and storage in *Phaeodactylum* strain Pt4.

## Introduction

We have to face decreasing resources in fossil fuels in the next few decades leading to the demand for sustainable production of alternative fuels [1]. Biofuels may contribute to closing this gap. However, they are primarily produced from crop plants resulting in the debate “food vs. fuel” [2]. That is why microalgae arouse interest. Microalgae can be grown on non-arable land with waste or seawater and therefore they do not compete with food crops [3]. Furthermore, it was suggested that the yield of oil per area is much higher in comparison to crop plants [4]. Among all phytoplanktonic microalgae, diatoms are most productive in carbon fixation. They accumulate lipids at least in the same amount or even more in comparison to other microalgae and moreover they are quite flexible to changing conditions [5]. That makes diatoms promising candidates for the production of biofuel [6, 7].

*Phaeodactylum tricornutum* is a model organism for diatoms and at least ten different accessions are available in the algae collections worldwide (Pt1 –Pt10) originating from all over the northern hemisphere. Although *P*. *tricornutum* is a pleiomorphic organism, most of the accessions occur naturally in the fusiform shape and have been assigned to four different genotypes, which are based on the genetic differences in sequences of the fast-evolving internal transcribed spacer 2 [8, 9]. Nevertheless, all accessions show a sequence identity of 97%–100% [8].

While most of the laboratories work with the sequenced strain Pt1, this work deals with Pt4 (UTEX 646). Accession Pt4 is quite special, because it is the only strain with genotype “B” [8]. This strain originates from the Island of Segelskär (Finland) in the Baltic Sea, where the salinity is only on average about one fifth in comparison to the oceans (Finnish Meteorological Institute, http://en.ilmatieteenlaitos.fi/seas). Therefore, this strain should allow growth under low salinity, which makes it suitable for production sites not located in coastal areas, where saline water is less available or wastewater may be the prevalent resource.

In the last years, several studies addressed lipid analysis of green algae and eustigmatophytes [9–14]. The knowledge gained from these analyses cannot be adopted for diatoms in general, since algae have evolved very differently and therefore lipid metabolism is most likely different in the algae kingdom, too [15]. Furthermore, diatoms are heterokonts, as their metabolic processes and pathways evolutionary originate from plants and animals [5, 15]. The reason for this is that diatoms originate from a secondary endosymbiosis between a heterotrophic eukaryote and a red alga [5, 16, 17]. The aim of this work was to provide more insights to the complex processes in lipid metabolism and its interaction with central metabolism caused by nitrogen (N)-depletion in the strain Pt4.

Like plants, algae in general exhibit two main classes of lipids: non-polar lipids (NL; e.g. acylglycerols like triacylglycerol (TAG) and its precursor diacylglycerol (DAG)) and polar lipids (glyco- and phospholipids (GLs and PLs)) [18]. TAGs serve as energy and carbon storage compounds, while polar lipids function as both structural constituents of membranes and intermediates for lipid synthesis. The GL are the main constituents of plastidic membranes and consist of monogalactosyldiacylglycerol (MGDG), digalactosyldiacylglycerol (DGDG) and sulfoquinovosyldiacylglycerol (SQDG). PLs primarily consist of phosphatidylcholine (PC), phosphatidyethanolamine (PE) and phosphatidylglycerol (PG). They are thought to be the major PLs, whereby phosphatidylserine (PS) and phosphatidylinositol (PI) are less present. Phosphatidic acid (PA) also belongs to the PLs. It represents a precursor in the biosynthesis of other lipids including DAG and serves as a signaling molecule. PLs are components of the extra-plastidic membranes, except PG, which is located in the thylakoid membrane. In addition, algae harbor in contrast to flowering plants betaine lipids in their membranes and three types of betaine lipids are known to occur in microalgae [19]. However, data on occurrence of betaine lipids of *Phaeodactylum* are ambiguous, since diacylglyceryl-hydroxy-methyl-N,N,N-trimethyl-β-alanine (DGTA) was detected as well as diacylglyceryl-N,N,N-trimethylhomoserine (DGTS) [9, 19, 20]. Betaine lipids have structural similarities to PLs. In contrast to PLs, their head group is not linked via a phosphate group to the DAG moiety but with an ether bond instead. It has been suggested that betaine lipids may have similar functions or even overtake the function of PLs under phosphate limiting conditions [21]. In plants, PC serves as a structural membrane lipid as well as central intermediate for lipid metabolism. The green algae *Chlamydomonas reinhardtii* contains no PC but DGTS [22]. It was supposed that DGTS is unlikely to be a precursor for other lipids like TAG as is PC in plants. The reason is the stronger ether linkage in DGTS in comparison to the phosphate linkage in PC. *Phaeodactylum* is known to contain both lipid classes [21]. This may suggest that lipid metabolism in diatoms presents an intermediate between lipid metabolism in green algae and higher plants [15, 18, 23].

The accumulation of triacylglycerols (TAG) in algae occurs mainly under stress conditions, especially phosphorous and N-depletion [15]. Under these conditions, the biomass production comes to a standstill and the amount of free amino acids and proteins are reduced massively [17, 24]. In some diatom species, this reduction is accompanied by an accumulation of citrate [25]. It was suggested, that slow growing diatoms have an enhanced capability to adapt to low nitrate conditions [25]. For an industrial production, TAG accumulation with continuous biomass production is the most favorable scenario and this may only be achieved by genetic optimization [26].

In order to achieve this goal, the basic understanding of these complex processes are even more important as started for diatoms [25] and in particular *Phaeodactylum* [9]. Therefore, the changes in lipid composition and amounts as well as changes in basic metabolism under N-depletion of *Phaeodactylum* strain Pt4 were analyzed by lipidomics as well as metabolite profiling and fingerprinting.

## Material and Methods

### Culturing

The *Phaeodactylum tricornutum* strain UTEX 646 was obtained from the UTEX Culture Collection of Algae. The cultures were grown in the seawater medium RSE (according to [27]). The N-depleted medium was produced by omitting KNO_3_. The cells were cultivated in columns (volume of 1 l) that were supplied with 2% CO_2_. The precultures were diluted in several cycles with fresh medium for avoiding nutrient depletion. Measurement of the growth parameters chlorophyll a (Chl*a*) and dry weight content were performed as described [28]. The cultures for the N-deplete growth kinetic were washed with N-free medium before inoculating the main culture. The Chl*a* content of the inoculated control cultures represented just one third (~5 mg/l) of the starvation cultures in order to allow exponential growth conditions for the following 7 days. A higher Chl*a* content would have led to an early shortage of nutrients resulting in stationary growth of cells. For the replete growth curve, the cells were illuminated with 120 μmol m^-2^ s^-1^ (referred as normal light), whereas the light intensity for N-deplete growth kinetics was 170 μmol m^-2^ s^-1^ (referred as normal light) respectively 350 μmol m^-2^ s^-1^ (referred as high light). For each condition and time point three biological replicates were generated and used for all following analysis. For the nitrogen-replete conditions, samples were collected during the growth curve (days 0, 2, 5 and 7 following inoculation), to reach the stationary phase (7 d). Under nitrogen-deplete conditions, the cultures enter the stationary phase earlier. Therefore, two relatively close time-points (days 2 and 3) were included to examine any possible alterations in lipid classes and metabolites at the earlier stages of nitrogen starvation. Preliminary work had indicated that longer cultivation in the nitrogen-free medium did not result in any further significant increase in biomass and TAG accumulation under the described experimental conditions. The Chl*a* as well as the dry weight content were measured parallel to taking samples from the cultures. This means at days 0, 2, 5 and 7 for replete conditions and days 0, 2, 3 and 6 for N-deplete conditions.

### Lipid extraction

For lipid extraction, 10 mg of lyophilized material was used. The lipids were extracted according to [29]. Before extraction, internal standards for quantification were added: Di-17:0-DAG and Tri-17:0-TAG were obtained by Sigma-Aldrich (Munich, Germany). Di-17:0-MGDG standard was provided by Prof. E. Heinz (Hamburg, Germany). Di-17:0-PC, Di-17:0-PE, Di-17:0-PG, Di-17:0-PS, 17:0-Lyso-PC, 14:0-Lyso-PE, 14:0-Lyso-PG and D_9_-DGTS were ordered at Avanti Polar Lipids (Alabaster, Alabama, USA). NLs, GLs and PLs were separated via solid phase extraction (Strata SI-1 Silica, 500 mg/6 ml, Phenomenex, Aschaffenburg, Germany). After lipid extraction, the samples were solved in 1 ml chloroform and loaded on the columns [30]. The NLs were eluted with 14 ml chloroform, the GLs with 15 ml acetone/isopropanol (9:1, v/v) and the PLs with 15 ml methanol/acetic acid (9:1, v/v). After evaporation of the single fractions, the dried samples of each lipid class were solved in 300 μL chloroform/methanol (1:2, v/v) with 5 mM ammonium acetate. The samples were either directly used for the analysis by direct infusion-nano electrospray ionization-tandem mass spectrometry (DI-nanoESI-MS/MS) or the samples were transmethylated with sodium methoxide to create fatty acid methyl esters (FAMEs) for gas chromatography analysis with flame ionization detection (GC-FID) [31].

### Central metabolite extraction

For the metabolite profiling measurements by GC-mass spectrometry (MS) analysis, 10 mg of flash frozen and lyophilized algae material were ground to a fine powder using a beatmill (Retsch, Haan, Germany) and glass beads (5 mm, Carl Roth, Karlsruhe, Germany). The polar fraction was extracted and derivatized with 30 μl methoxyamine hydrochloride and 60 μl N-methyl-N-(trimethylsilyl) trifluoroacetamide (MSTFA) as previously described to transform the metabolites into their methoxyimino (MEOX)- and trimethylsilyl (TMS)- derivatives [32]. Ribitol (Sigma-Aldrich, Munich, Germany) was used as an internal standard.

### GC-FID and GC-MS

For FAME analysis, an Agilent 6890 gas chromatograph with a capillary DB-23 column (30 m * 0.25 mm; 0.25 μm coating thickness; Agilent Technologies, Waldbronn, Germany) was used. As mobile phase helium was used with a constant flow rate of 1 ml/min. The injection was performed in the split mode at 220°C. The temperature gradient was 150°C for 1 min, 150–200°C with 4 K/min, 200–250°C with 5 K/min and hold 250°C for 6 min. The chromatograms were analyzed with the software ChemStation (Agilent Technologies). For validation, the different retention times of the FAMEs were compared to a FAME standard (F.A.M.E. Mix C4 – C24; Sigma-Aldrich, Munich, Germany). The identity of the FAMEs was verified using GC-MS (Agilent 5973 network mass selective detector connected to an Agilent 6890 gas chromatograph (Agilent, Waldbronn, Germany) with a capillary DB-23 column (30 m x 0.25 mm, 0.25 μm coating thickness, Agilent Technologies). In addition to the system settings described above, electron energy of the mass spectrometer was set at 70 eV with an ion source temperature of 230°C and a transfer line temperature of 260°C. Ions were detected in scan mode within a range of *m/z* = 50 – 650. GC-MS data were analysed using the Agilent MSD ChemStation data analysis software (Agilent Technologies).

For the measurement of central metabolites, the inlet temperature was set to 230°C and the temperature gradient applied was 50°C for 2 min, 50 – 330°C at 5 K/min, 330°C for 2 min. Here, a transfer line temperature of 330°C was used. Spectra were recorded in the range of *m/z* = 71 – 600. Masses used for quantification of the individual metabolites are depicted in S1 Table. In the case, derivatization of metabolites led to several peaks of interest, their abundance was added based on the total ion count (TIC) of the individual analytes.

### Analysis of molecular lipid species

The analysis of the molecular lipid species was performed with DI-nanoESI-MS/MS using a TriVersaNanoMate® equipped with an ESI Chip^TM^ (AdvionBioSciences, Ithaca, USA). For negative and positive mode, voltage was applied to 1.25 kV, the gas pressure was set to 0.2 psi and 0.1 psi, respectively. After infusion, the sample was analyzed by hybrid triple quadrupole/linear ion trap tandem mass spectrometry using QTRAP4000 (AB Sciex, Framingham, USA) equipment. GLs and PLs were analyzed with multiple reaction monitoring (MRM) in the negative ion mode. The Q1 and Q3 setups were defined by the sum of the predicted masses of [M-H+CH_3_CO_2_H]^-^ of MGDG, DGDG, DGTS and PC, or [M-H]^-^ for SQDG, PA, PE, PG, PI and PS, and all possible combination of FAs bound to the corresponding lipid. The NLs were measured with neutral loss scanning [32]. The acyl-chain fragments for Q3 setups are listed in Table 1 and the analysis was done with the software LipidView™ (AB Sciex).

**Table 1:** Acyl chain fragments for MRM and NL scanning.

Acyl chain fragment	m/z*	Acyl chain fragment	m/z*
**14:0**	227.2	18:1	281.2
**16:4**	247.2	18:0	283.3
**16:3**	249.2	20:5	301.4
**16:2**	251.2	20:4	303.5
**16:1**	253.2	20:3	305.6
**16:0**	255.2	20:0	311.7
**17:0**	269.2	22:6	327.5
**18:4**	275.4	22:5	329.5
**18:3**	277.2	24:0	367.4
**18:2**	279.2		

*corresponding ammonium adducts used for NL scanning

### Acyl-CoA analysis

Acyl-CoAs were extracted from 8 mg of freeze dried powdered diatom as described by [33]. The analysis was performed with Ultra High Performance Liquid Chromatography (UHPLC)-MS/MS using an Agilent 1200 LC system (Agilent Technologies) equipped with a Gemini C18 column (150 mm x 2 mm, 5 μm, Phenomenex, Torrance, USA) and a QTRAP4000 tandem mass spectrometer (AB Sciex, Framingham, USA) in positive ion mode and multiple reaction monitoring mode as described by [34]. For the identification and calibration, standard acyl-CoA esters with acyl chain lengths from C14 to C20 were purchased from Sigma-Aldrich as free acids or lithium salts.

### Metabolite fingerprinting

10 mg of lyophilized and homogenized algae material was used for extraction according to [29] with methyl-*tert*-butyl ether. Metabolite of the polar extraction phase were solved in methanol/acetonitrile/water (10:10:120, *v/v/v*), centrifuged and analyzed by Ultra Performance Liquid Chromatography (UPLC, Waters, Milford, MA, USA) coupled with a photo diode array (PDA, Waters) detector and an orthogonal time-of-flight mass spectrometer (TOF-MS, LCT Premier, Waters). UPLC-ESI-TOF-MS analysis and data deconvolution was performed as described in [35]. Data processing and data mining was carried out with the toolbox MarVis (MarkerVisualization, http://marvis.gobics.de, [36, 37])

## Results

### Cultures stop to grow under N-deplete conditions

**Fig 1:**
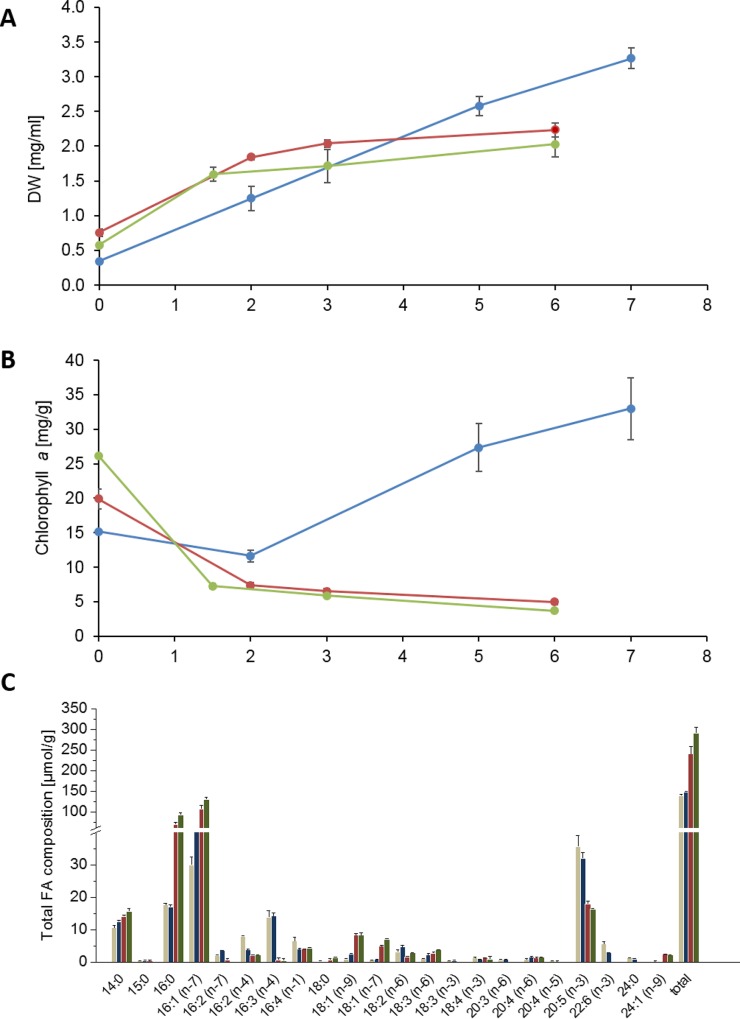
Growth parameters and total fatty acid composition of *Phaeodactylum tricornutum* control cultures under normal light (blue) or with N-depletion under normal light (red) or high light (green). In (A) the dry weight content [mg/ml] is depicted and in (B) the chlorophyll *a* amount in relation to the dry weight [mg/g]. Samples were taken at days 0, 2, 5 and 7 for N-replete growth and at days 0, 2, 3 and 6 for N-depleted growth. These samples were used for determination of growth parameters as well as for lipid and metabolite analysis. The total FA composition in μmol/g is displayed in (C) showing day 0 and the last time point of each condition. Day 0 comprises the mean of all conditions (beige). Day 7 of replete conditions is shown in blue, day 6 of N-deplete with normal light in red and day 6 of N-deplete with high light in green. Data are mean values of 3 biological replicates, for the comprised time point 0d, 4 biological replicates were used. Error bars indicate standard deviation.

The aim of this work was to gain information on the effects of N-depletion in general as well as N-depletion under normal and high light in the strain Pt4 that can grow in low salt or wastewater-derived media. To compare the effects, growth kinetics under control conditions (replete medium, normal light; Fig 1, blue lines or bars), N-depletion (normal light; Fig 1, red lines or bars) and N-depletion in combination with high light (Fig 1, green lines or bars) were recorded in the presence of 2% CO_2_. Samples were taken within the first 6 days (for N-depleted cultures) and 7 days (for control cultures), respectively, after inoculation with an exponential growing preculture. The cells cultivated in replete medium continued in logarithmic growth while the cells cultivated in N-deplete medium stopped growing after 2 days (Fig 1A and 1B). Decreasing Chl*a* content related to biomass suggested protein degradation under N-depletion (Fig 1B, red and green lines vs. blue line). Interestingly, there were just minor differences between normal and high light (Fig 1B, red vs. green line).

### Only C16 fatty acids increase upon N-depletion

The main FA in total lipid extracts of Pt4 were 14:0, 16:0, 16:1((n-7), x:y(n-z) denotes a fatty acid with x carbons and y double bonds in position z counting from the methyl end), and 20:5(n-3) (Fig 1C). Overall, the total FA profile is low in C18 FAs and in 22:6(n-3) and showed only minor changes upon N-repletion in comparison to the samples of the starting point (Fig 1C, blue bars vs. beige bars). However, under N-depletion the total FA content was almost doubling from about 140 μmol/g to 240 μmol/g at normal light and to 290 μmol/g at high light respectively on 6 d, meaning the total FA content increased by 70% under normal light and 107% under high light. The total amount of 16:0 and 16:1(n-7) was increasing fourfold and twofold respectively. In contrast, the proportion of 20:5(n-3) was reduced by two thirds whereas 22:6(n-3) could be hardly detected. Generally, the differences in the FA content under N-depletion under normal light and high light seem to be small (Fig 1C, red and green bars vs. beige bars).

### MGDG and DGTS are the major lipid classes that are degraded upon N-depletion

According to the total FA profile, there were clear changes due to N-starvation or depletion. Next, total FA distribution in NLs, GLs and PLs was analyzed (S1 Fig). Under N-depletion, the amount of 16:0 was doubling in NLs and GLs (S1A and S1B Fig) and 16:0 and 16:1(n-7) were the main FAs of NLs (S1A Fig). The GLs fraction was reduced in 16:2(n-7), 16:2(n-4) and 16:3(n-4) (S1B Fig) whereas C18 FAs and 22:6(n-3) were predominantly found in the PL fraction (S1C Fig). On the other hand, the amount of 20:5(n-3) was mainly increasing in PLs (1.7 fold and 1.5 fold respectively, comparing replete and N-depleted conditions, S1C Fig) while it was 1.6 fold decreasing in NLs (S1A Fig).

To get a deeper insight into the biosynthesis of the different lipid classes, we analyzed twelve lipid classes at the molecular species level by DI-nanoESI-MS (S1 Table). Except PA and PI, which could be estimated as relative amounts only, all other analyses represent quantitative data. S2 Fig shows the sum of all intact molecular species detected for each lipid class. The main lipid classes of Pt4 in all conditions were the betaine lipid DGTS, the glycolipid MGDG and the phospholipid PC. Under replete conditions DGTS had the highest amount with 48 μmol/g, followed by MGDG (31.1 μmol/g) and PC (6.1 μmol/g) after 7 d (S2 Fig, blue bars). Under N-starvation, TAG showed a massive increase from 0.1 to 11.3 μmol/g during starvation and DAG was quadrupling its amounts (S2 Fig, red bars). In contrast, MGDG was greatly reduced from 31.1 to 6.8 μmol/g showing the strongest change compared to other plastidial lipids. By taking the standard deviation into consideration, DGTS seemed to be the only lipid that decreased only under normal light to about one third of its initial amount. In addition, PG, PE and PI decreased under N-limitation. Together, the largest changes between the lipid classes were an increase in TAG and the decreases of DGTS and MGDG, suggesting that these two lipid classes may serve as the main FA sources or precursors for TAG formation. Besides these lipid species, we detected the associated lysolipid species as well (S3 Fig). It is quite striking that all lysolipid species showed the same pattern, unlike the intact lipid species. Under N-replete conditions the amount of lysolipid species were increasing during exponential growth up to 5 d and decreased afterwards in the late exponential phase at 7 d (blue bars). By N-depletion, the amount of lysolipid species were massively reduced and MGMG, DGMG, LPG and MGTS could hardly be detected. S4 Fig summarizes both values at 7 d replete (blue bars) and 6 d N-deplete, respectively (red and green bars). Together, the amount of TAG increased on the expense of MGDG and DGTS suggesting that the later lipid classes may serve as TAG precursors.

### DGTS molecular species most likely serve as precursors for TAG formation upon N-depletion

**Fig 2:**
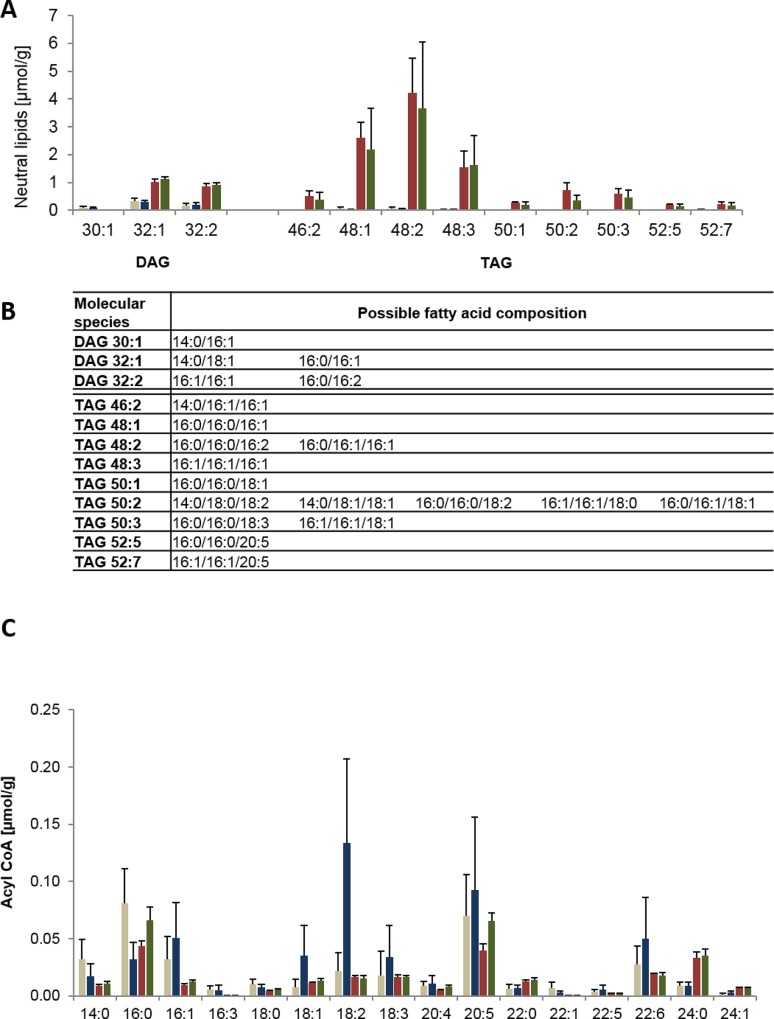
DAG and TAG molecular species as well as acyl-CoA pool under replete and N-deplete conditions. The amounts of the molecular species of diacylglycerol (DAG) und triacylglycerol (TAG) are displayed with a threshold of 0.1 μmol/g in (A). These data were produced by neutral loss scanning which allows the determination of the sum (number of carbon atoms and of double bonds) of two (for DAG) or three FAs (for TAG) but not always the elucidation of the distinct fatty acid composition of the molecular species. Therefore, the possible fatty acid composition of the different molecular species are given in (B). (C): The diagrams show the acyl-CoA pool as comparison of day 0 (beige) and the last day of the growth kinetic of growth under replete conditions (blue), N-depletion with normal light (red) and N-depletion with high light (green). Data are mean values of 3 biological replicates, for the comprised time point 0d, 9 biological replicates were used. Error bars indicate standard deviation.

The major aim of this study is to understand TAG formation in order to later increase its production. Therefore, the biosynthetic origin of the TAG molecules formed was of main interest and the molecular species composition of TAG was analyzed next. As a direct precursor of TAG, DAG was analyzed first. It consisted of two main species: 32:1 and 32:2 (Fig 2A) and during N-starvation the amount of these species increased about 4–6 fold. However, the absolute identity of these two lipid species could not fully resolved: 32:1 consisted either of 14:0/18:1 or 16:0/16:1 or represents a mixture of both (Fig 2B). Similarly, 32:2 may consist of 16:1/16:1 or 16:0/16:2 or again of both species.

The molecular TAG species consisted primarily of the C16 FA-containing species 48:1, 48:2 and 48:3 (Fig 2A and 2B). Moreover, only 20:5(n-3) and no 22:6(n-3) was detected in TAG species and only one 20:5(n-3) was always combined with two C16 FA.

Since the n-3 FAs 20:5(n-3) and 22:6(n-3) were underrepresented in the NL fraction, the question arose whether the acyl-CoA pool was a limiting factor during N-starvation. In contrast to the FA profile, the main acyl-CoA species were 16:0, 16:1, 20:5, 22:6 and 24:0 (Fig 2C). This was remarkable since 22:6 and 24:0 could be hardly detected as in the total FA profile. Thus, there seems to be no shortage of both n-3 FAs for NL biosynthesis, but their incorporation may rather be limited by the substrate specificities of the enzymes involved.

**Fig 3:**
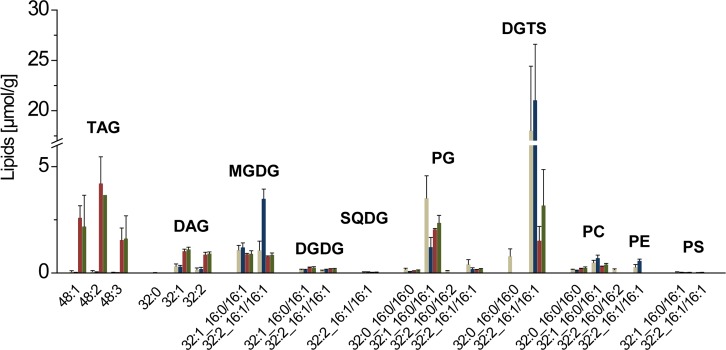
Main molecular lipid species that may be relevant for TAG synthesis. The diagrams show the comparison of day 0 (beige) and the last day of the growth kinetic of growth under replete conditions (blue), N-depletion with normal light (red) and N-depletion with high light (green). Data are mean values of 3 biological replicates, for the comprised time point 0d, 9 biological replicates were used. Error bars indicate standard deviation.

A similar FA backbone as in TAG was not only detectable in its direct precursor DAG, but also in the GL and the PL fraction. Therefore, all lipid classes were examined in detail for molecular species, harboring two FAs that matched the pattern of the main TAG species (48:1, 48:2, 48:3, cf. Fig 2A and 2B). For this, the major molecular species of 0d (Fig 3, beige bars; S2 Table) were compared with 7d replete conditions (Fig 3, blue bars) and 6d N-deplete conditions (Fig 3, red and green bars; S2 Table). The TAG-relevant species of DGDG, SQDG, PG, PC, PE and PS were not significantly altered during N-depletion. However, MGDG_32:2 (3.5 to 0.8 μmol/g) and DGTS_32:2 (21 to 1.5 μmol/g) decreased under N-shortage (Fig 3, blue vs red bar; S2 Table). Comparing the absolute amount of both species, reveals DGTS_32:2 as the most promising species that directly feed into TAG biosynthesis.

### Metabolite analysis reveals an induction of fermentation and Calvin Cycle intermediates after N-depletion

Next, the same samples were analyzed by metabolite fingerprinting and a data set of 935 high quality features with a false discovery rate (FDR) < 10^−5^ was obtained from the polar extraction phase. The scatter plot of the Principle Component Analysis (PCA, S5 Fig) showed that all N-depleted samples clustered together, while the replete samples are widely spread. The former behavior may be explained with the observation that these cultures stopped growing in contrast to the replete cultures (Fig 1A). The light intensity seems to generate slight differences in the metabolic content of the N-depleted algae. However, the metabolic adaption of the algae to the N-starvation seems to be completed already in the 3 d-old cultures.

**Fig 4:**
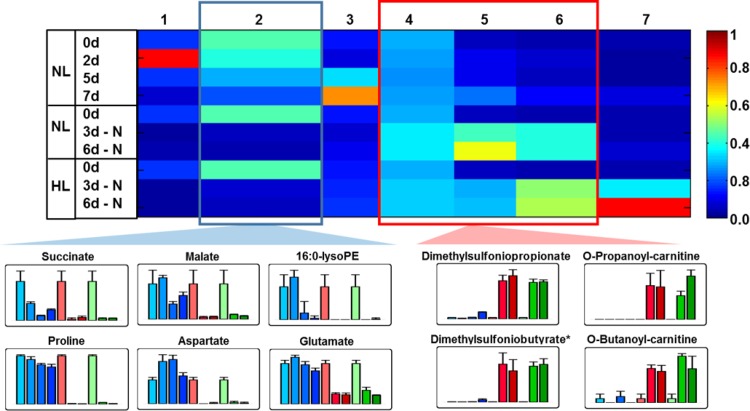
Metabolite fingerprinting analysis of *Phaeodactylum tricornutum* under replete and N-deplete conditions. *Phaeodactylum tricornutum* cultures grown under replete condition and normal light (NL), or grown under N-depleted conditions (-N) and normal light (NL) or high light (HL) were harvested at the indicated time points and extracted by two phase partitioning. The fingerprint of metabolites of the polar extraction phase was generated by UPLC-TOF-MS analysis. A subset of 935 high-quality features (FDR < 10^−5^) derived from the positive as well as the negative ionization mode were used for clustering and visualization by means of one-dimensional self-organizing map (1D-SOM, http://marvis.gobics.de). Prototype 2 (blue frame) represents features with reduced relative amounts under N-depleted conditions, while features combined in prototype 4–6 (red frame) are enriched under N-depletion. Horizontal and vertical dimensions correspond to prototypes and experimental conditions, respectively. The heat map colors represent average intensity values according to the color map on the right-hand side. The width of each prototype column is proportional to the number of marker candidates assigned to this prototype. Bar plots show mean values with standard deviations of 3 biological replicates for prominent metabolite markers of the selected clusters. The first 4 bar plots (light to dark blue) show the relative amounts of the compounds for replete conditions (0, 3, 5, 7d), while the next bar plots show the data for N-deplete conditions (0, 3, 6d) under normal light (light to dark red) or high light (light to dark green). The identity of the indicated compounds was confirmed by high resolution MS^2^ experiments. Visualization was applied using VANTED 2.1 software [39].

Thereafter, the intensity pattern of the 935 high quality features were clustered by means of an one-dimensional self-organizing map (1D-SOM) using the data mining and visualization tool MarVis Cluster [36, 37]. The number of 7 clusters has been chosen to represent best the feature pattern in respect to the ten experimental conditions (Fig 4, see also S3 Table). Cluster 2 and 4–6 of the 1D-SOM representation show an inverse pattern in respect to the N-depletion. While cluster 2 represents features being reduced under N-starvation, cluster 4–6 contain features, which accumulated under this condition. The exact mass information of the features of the selected clusters was used for data base search and metabolite set enrichment analysis (MSEA) by MarVis Pathway [36]. The metabolite classes most obviously depleted under N-starvation (Fig 4, cluster 2) were amino acids, lyso-PLs and TCA cycle intermediates, as can be exemplary seen by the bar plot presentation of selected metabolites. Additionally fragments of chrysolaminarin consisting of 7 or less hexose units (putatively identified by MS/MS, S3 Table) as a linear polymer of β(1→3) and β(1→6)-linked glucose units, were found in cluster 2. Chrysolaminarin serves in photosynthetic heterokonts as a storage polysaccharide and seems to be consumed under N-depletion [5, 38].

**Fig 5:**
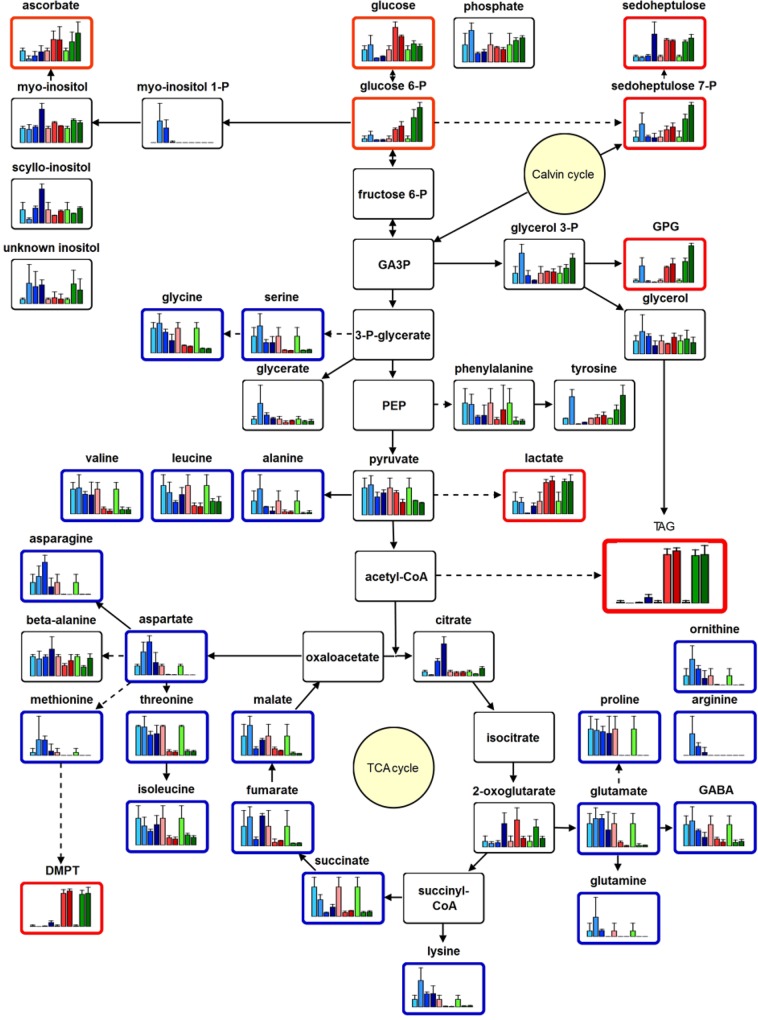
Profiling analysis of the central metabolites of *Phaeodactylum tricornutum* under replete and N-deplete conditions. *Phaeodactylum tricornutum* cultures were analyzed by metabolite profiling. Bar plots show mean values with standard deviations of 3 biological replicates for prominent metabolite markers of the selected clusters. The first 4 bar plots (light to dark blue) show the relative amounts of the compounds for replete conditions (0, 3, 5, 7d), while the next bar plots show the data for N-deplete conditions (0, 3, 6d) under normal light (light to dark red) or high light (light to dark green). The identity of the indicated compounds was confirmed by high resolution MS^2^ experiments. Pathway visualization was applied using VANTED 2.1 software [39].

Two of the features in cluster 4–6 showing a strong accumulation upon N-starvation are S-containing metabolites: dimethylsulfoniopropionate (DMSP) and the tentatively identified dimethylsulfoniobutyrate (S6 Fig). In contrast to the well-known DMSP, dimethylsulfoniobutyrate was to our best knowledge so far not described in any organism. The high resolution MS/MS spectra of both compounds showed a neutral loss of the S(CH_3_)_2_-moiety (m/z 62.0182), which led to the fragment of *m/z* 73.0282 for the remaining C3-acyl moiety [40] as well as of *m/z* 87.0440 for the C4-acyl moiety (S6 Fig). DMSP is known to replace the N-containing osmolyte proline under N-starving conditions in algae to enable proper osmoadaptation [17, 41]. Beside these compounds, also the very simple structured S-containing compound methylsulfate is enriched after N-depletion (S6 Fig). In addition, short acyl chain carnitines, tentatively identified as propanoyl-carnitine and butanoyl-carnitine, accumulated strongly after N-depletion (Fig 5 and S6 Fig). High resolution MS/MS analyses in the positive ionization mode confirmed the identity of propanoyl-carnitine by detecting signals of the trimethylamine-moiety (*m/z* 60.0807, NH(CH_3_)_3_^+^) and the butyryl-residue (*m/z* 85.0281, C_4_H_5_O_2_) as diagnostic fragments for acyl-carnitines [42] (Metlin data base: METLIN ID52 for Carnitine).

In addition to the non-targeted metabolite fingerprinting analysis, primary metabolites were measured by metabolite profiling (S4 and S5 Tables). These results confirmed a rapid depletion of the amino acids and for the intermediates of the TCA cycle after 3 d of N-deprivation with the exception of 2-oxo-glutaric acid, which increased after 3 d but depleted three days later (Fig 5).

The highest absolute increase of a primary metabolite, analyzed by GC-MS analysis, was sedoheptulose, a monosaccharide with seven carbon atoms (heptose). This is a metabolite most likely originating from the Calvin Cycle. Sedoheptulose was present in all samples. Whereas the compound accumulated at the latest time point of replete growth (7 d), it was already increased at 3 d under N-depleted conditions. Sedoheptulose may have been formed by a so far unknown phosphatase from sedoheptulose-7-phophate, as this metabolite was also increased. Sedoheptulose can be considered a very uncommon metabolite in algae especially in such a strong abundance, much stronger even than glucose. In addition, the accumulation of lactate was observed.

## Discussion

The aim of this work was to provide insight into lipid metabolism and its interaction with central metabolism caused by nitrogen (N)-depletion in the *Phaeodactylum* strain Pt4. We focused on this *Phaeodactylum* strain, because of its ability to grow in medium with low salinity and therefore being suited for wastewater cultivation strategies. Although the analysis of lipid remodeling triggered by stress conditions, especially N-starvation, is in focus of algae research [9, 17, 43, 44], analysis of lipid molecular species under stress conditions, acyl-CoA profiling together with metabolome analysis are yet missing for most of the *P*. *tricornutum* strains.

### Different microalgal strains display different TAG accumulation capacities

A comparison of nine different microalgae strains (freshwater and marine strains) showed a wide range TAG content of app. 5% to over 40% of dry weight under N-depletion [43]. Even within the species *Phaeodactylum*, the TAG accumulation ability varies a lot [9]. This suggests for different regulatory principles of lipid biosynthesis or even different ways how these pathways are being organized in different algae.

### N-depletion causes an arrest in biomass production

As long as nutrients were available in sufficient amount, *Phaeodactylum* Pt4 grew exponentially, evident by an increasing number of cells and the Chl*a* content (Fig 1A and 1B). Under nitrogen depletion, however, biomass production came to a standstill as has been already described for another *Phaeodactylum* strain [45]. Chlorophyll as well as photosynthetic complexes are reduced due to the N-restriction what affects photosynthesis and cell division. This growth arrest is well established for other algae species like *Chlamydomonas reinhardtii*, *Hematococcus pluviales* and *Nannochloropsis gaditana* [10, 46, 47]. Besides N-depletion under normal light, we analyzed also N-depletion under high light. It has been described that combined N-limitation and high light together cause a more severe stress response than N-starvation alone [48]. However, this was neither visible for biomass nor for TAG production in Pt4 (Fig 1), indicating that carbon allocation to biomass and TAG was restricted by further increase in light intensity (meaning higher incident light per cell).

### N-depletion leads to an increased C16 FA content

Under N-depletion, the amount of total FAs was almost doubling (Fig 1C, red and green bars). Especially 16:0 and 16:1 were increasing, while polyunsaturated C16 FAs, 20:5(n-3) and 22:6(n-3) were decreasing. This correlates with the finding that the unsaturation of FAs is decreasing under N-starvation in several microalgae and particularly marine species show a decrease in very long chain PUFAs [43]. These changes in Pt4 predominantly reflect changes in the NL profile (S1A Fig) and 20:5(n-3) is transferred from GLs to PLs (including DGTS). This is one of the main differences to the *Phaeodactylum* strain Pt1 that has been sequenced and is in focus of *Phaeodactylum* research. Glycerolipid remodeling was investigated under N-limitation in Pt1 [9], but the acyl-CoA pool and changes in the molecular species caused by starvation were not analyzed. In Pt1, 20:5(n-3) is increased in TAG and decreased in MDGD under N-starvation, while its amount was not changed in PLs. Another *Phaeodactylum* strain (UTEX640) was analyzed for its total FA profile. This strain also displays an increase in 16:0 and 16:1 and a decrease in 16:2, 16:3 and 20:5 like the strain Pt4 [43]. Both studies can hardly detect 22:6(n-3), but we could clearly detect 22:6(n-3) predominantly in PLs (including DGTS) (S1C Fig).

The main lipids in Pt4 were DGTS, MGDG, PC and PG under normal growth conditions. Contrary to Pt4, Pt1 showed a higher amount of SQDG and a much lower amount of the betaine lipid DGTA [9]. N-starvation caused in both strains a reduction in MGDG, SQDG and PG and this reduction was much more pronounced in Pt4 under conditions examined in this work (S2 Fig). This reduction is caused by a downregulation of photosynthesis, which is accompanied by a decrease of thylakoid membranes [10].

In higher plants, no betaine lipids are present, whereas in *Chlamydomonas* DGTS was detectable but no PC [49]. However, both lipids were present in *Phaeodactylum* Pt4 (S2 Fig). Since DGTS and DGTA have the same mass and we were not able to detect the absence of a *m/z* 87-fragment in the MS/MS spectrum of the betaine lipid species as reported before [9, 50], we decided to assign this lipid class to be DGTS. However, we are aware that only a nuclear magnetic resonance experiment of the isolated lipid fraction can unambiguously clarify the situation. In Pt4, the overall amount of DGTS and here especially those species harboring C16 FAs were decreasing due to N-depletion. Therefore, we conclude that primarily these lipids seem to be feed into TAG (Fig 3). In Pt1, TAG formation was not only analyzed for N-starving conditions, but for phosphate starvation, too. In the former situation only MGDG decreased, while the amounts of PC and DGTA are influenced by phosphate limitations. Here, the amount of PC is strongly reduced and the amount of DGTA is increasing [9]. The increase in non-phosphorous lipids like SQDG and DGTA is a well described reaction to cope with limiting phosphate concentrations in the environment [51], and even *Phaeodactylum* is known to react very flexible to environmental changes [5].

### Analysis of lipid molecular species identifies DGTS as major source of TAG formation

A central aim of this study was to identify the precursors of TAG in Pt4 in order to improve its productivity. To address this question, the underlying hypothesis of this research was that the limiting step in TAG production is catalyzed either by acyl-CoA:diacylglcerol acyltransferases (DGAT) or phospholipid:diacylglcerol acyltransferases (PDAT) [52]. Therefore, the focus was on those molecular membrane lipid species that had a similar FA backbone as the TAG species newly formed upon N-starvation. In addition, the second component of TAG formation, the acyl-CoA pool, was analyzed. Under N-depleted conditions, TAG was mainly composed of 16:0 and 16:1, just minor species with one 20:5(n-3) were determined and the main TAG species were 48:1, 48:2 and 48:3 (Fig 2A). TAG profiling of different marine microalgae also revealed a high amount of C16 FA [44, 53]. In addition, in our study the precursor DAG primarily contained 16:0 and 16:1 and no 20:5(n-3) was detectable (Fig 2A). This suggests that the minor TAG-20:5(n-3)-species originated from DAG that is acylated with 20:5- CoA by either one of the four DGATs *P*. *tricornutum* [54].

Interestingly, there were differences in Pt1 in comparison to Pt4. Additionally to 16:1, there was also a remarkable increase of 20:5(n-3) in TAG. This difference suggests the occurrence of different or even additional acyltransferases that incorporate preferentially 20:5(n-3) into TAG, since the increase of 20:5(n-3) may have been caused by the additional observed increase in 20:5(n-3)-containing DAG species that have been detected in Pt1 under replete conditions [9]. Among all acyl-CoA species, 20:5-CoA was the main species in Pt4 as already described for normal growth conditions [55]. This amount was unchanged under N-depletion. Hence, there may be enough 20:5(n-3) available for the incorporation in TAG although the total amount of acyl-CoA was reduced by about one-half (Fig 2C).

Overall, in *Phaeodactylum* Pt4 the main TAG species are 48:1, 48:2 and 48:3 (Fig 2A). A search for related backbones within the molecular species of membrane lipids revealed that substantial amounts of MGDG, PG and DGTS harbored a similar FA pattern. However, only the reduction in MGDG_32:2 and DGTS_32:2 was dependent on N-starvation (Fig 3) and out of these two species only the decrease in DGTS_32:2 may be large enough to match the increase in TAG. This strongly suggests a conversion of primarily DGTS to TAG in PT4. However, it should be pointed out that an enzyme that converts DGTS into DAG has not been described yet and that only a transfer of FAs from this lipid via the action of a phospholipase A_2_ has been suggested [56].

**Fig 6:**
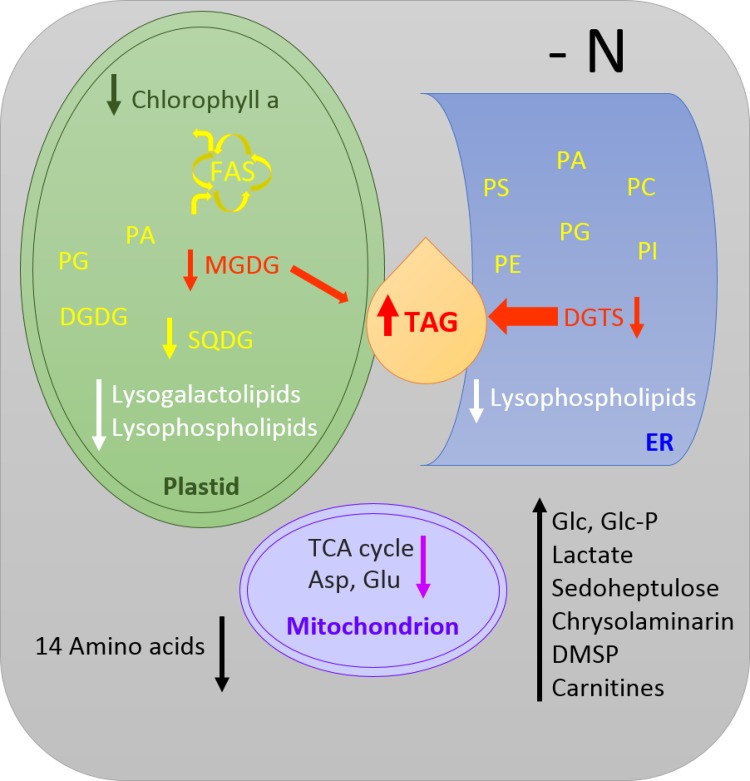
Summarizing scheme of changes of lipidome and metabolome under N-depletion. The scheme displays the main observations followed by N-depletion. Regarding lipid species (yellow) the main changes affect MGDG, DGTS and TAG, suggesting a major flux from DGTS towards TAG, but there are also evidence for a participation of MGDG and maybe SQDG. All lysolipid species are decreased. The Chl*a* content is decreasing which suggests together with a decrease of different amino acids towards protein degradation. In addition, the compounds of the TCA cycle are reduced. However, different sugar compounds as well as S-containing metabolites and carnitines are increased.

In other algal species, MGDG was proposed as another precursor for TAG synthesis [15]. In *Chlamydomonas* it was shown that a GL-lipase is required for TAG accumulation upon N-depletion, and for *Nannochloropsis*, a recycling from at least 20:5 to TAG was suggested [10, 20]. Since MGDG is the major lipid that is degraded in Pt1 upon N-depletion, there may be a similar mechanism in this *Phaeodactylum* strain [9] and we cannot exclude that in Pt4 FAs are released from MGDG and incorporated into TAG in a similar way. The data obtained in this study are summarized in Fig 6. Besides N-depletion, heat stress also causes TAG accumulation in *Chlamydomonas*. Lipid analysis suggested here again a remodeling of MGDG to DAG and TAG [56]. In addition, the authors suggested that the donor for the third acylation of DAG is DGTS or PE, since the corresponding lysolipid species were increased.

### N-depletion induces strong reprogramming of the primary and specialized metabolism

A detailed inspection of the central and specialized metabolism revealed strong changes within the first days after N-depletion (Figs 4 and 5). In general, almost all amino acids (despite of tyrosine) and three central intermediates of the TCA-cycle (succinate, fumarate and malate) were reduced in cultures, which were grown for 3 d under N-depletion. These data are in consistence with previous results for *Thalassiosira pseudonana* [14, 17] and *P*. *tricornutum* Pt1 [38, 57]. Diatoms adapt to N-starving conditions by reducing the amount of the N-containing metabolites and to recover rapidly, when nitrogen is available by a unique link between TCA-cycle, glutamine synthetase/glutamate synthase cycle and ornithine-urea cycle [57].

Interestingly, we found an accumulation of sedoheptulose upon N-starvation under our conditions in strain Pt4 so far neither been observed nor predicted to occur in Pt1 [58] (Fig 5). Such an accumulation has been previously described under CO_2_ enrichment for the CAM-plant *Kalanchoë pinnata* [59]. Similarly, it is possible that the presence of sedoheptulose in Pt4 is caused by the supplementation with CO_2_ as it is also present in the cells growing under replete conditions. However, sedoheptulose may also represent an intermediate carbon sink that can be quickly channeled back into the Calvin cycle.

By non-targeted metabolome analysis, compounds, which show an inverse pattern in respect to amino acids and TCA-cycle intermediates, were identified. These are mainly sulfur-containing metabolites like DMSP, its C4-analogue dimethylsulfoniobutyrate and methylsulfate (Fig 4 and S6 Fig). DMSP and dimethylsulfoniobutyrate are zwitterionic molecules, which may serve as compatible solute. DMSP is crucial for osmoprotection in marine algae especially under N-starving conditions, where the N-containing osmoprotectant proline is degraded [17, 41]. In addition, DMSP seems to be one key player of the sulfur cycle, because it functions as precursor of dimethylsulfide (DMS). The volatile DMS serves as link between the biogeochemical fixed sulfur in the ocean and the atmospheric sulfur [60].

In contrast to the strong depletion of the amino acids as N-containing metabolites, an accumulation of the N-containing short acyl chain carnitines propanoyl-carnitine and butanoyl-carnitine was detected after N-depletion (Fig 4 and S6 Fig). These quaternary ammonium compounds are positively charged and the carnitine-residues show structural similarities (quaternary amine coupled to a short acyl chain) to the zwitterionic osmolytes glycine betaine, trimethylammonium propionate and trimethylammonium butyrate. These compounds have been described as compatible solute in *Emiliania huxleyi* and *Prorocentrum minimum* [60]. Interestingly, trimethylammonium butyrate is homologous to carnitine, beside the lack of the hydroxy-group in the C2-position. This hydroxyl-group is used to bind the acyl-residues via an ester bond, which result in acyl carnitines. While long chain acyl carnitines may be part of the transport system for FAs into the mitochondrial matrix the function of the short chain acyl carnitines remain elusive. It is tempting to speculate that propanoyl-carnitine and butanoyl-carnitine could either serve as a new class of osmolytes or play a role in the interconversion of C3- and C4-building blocks, which enable *P*. *tricornutum* to adapt to episodic N-limitations by reprogramming the C- and N-metabolism very efficiently.

## Conclusions

This study focused on *Phaeodactylum* strain Pt4 (UTEX 646), because its ability to grow when saline water is less available or for wastewater cultivation strategies. Our data show an increase in neutral lipids during nitrogen-depletion and the molecular species composition of TAG suggests the betaine lipid DGTS as its precursor. Although, a contribution of the chloroplast galactolipid MGDG cannot be excluded. Other metabolites most obviously depleted under nitrogen-starvation were amino acids, lyso-phospholipids and tricarboxylic acid (TCA) cycle intermediates, whereas sulfur-containing metabolites as dimethylsulfoniopropionate, dimethylsulfoniobutyrate and methylsulfate increased upon nitrogen-starvation. Interestingly, the Calvin cycle may be regulated by sedoheptulose accumulation after nitrogen-depletion. Together the data provide now the basis for new strategies to improve lipid production and storage in *Phaeodactylum* strain Pt4.

## Supporting Information

S1 Fig **FA profile of neutral lipids (A), glycolipids (B) and phospholipids (C).** The diagrams shows day 0 and the last time point of each condition. Day 0 comprises the mean of all conditions (beige). Day 7 of replete conditions is shown in blue, day 6 of N-deplete with normal light in red and day 6 of N-deplete with high light in green. Data are mean values of 3 biological replicates, for day 0 9 biological replicates were used. Error bars indicate standard deviation.
(TIF)

S2 Fig Changes of different lipid classes during the growth kinetic. Absolute amounts (μmol/g) are given for triaclyglycerol (TAG), diacylglycerol (DAG), monogalactosyldiacylglycerol (MGDG), digalactosyldiacylglycerol (DGDG), sulfoquinovosyldiacylglycerol (SQDG), phosphatidylglycerol (PG), diacylglyceroltrimethyhomoserine (DGTS), phosphatidylcholine (PC), phosphatidyethanolamine (PE), and phosphatidylserine (PS). For phosphatidylinositol (PI) and phosphatidic acid (PA) the relative amounts are depicted. Samples were taken at 0d, 2d, 5d and 7d for N-replete conditions (blue, tones are getting darker with increasing days). Sampling for N-deplete conditions were done at 0d, 2d, 3d and 6d. Normal light is shown in red (tones are getting darker with increasing days) and high light in green (tones are getting darker with increasing days). Data are mean values of 3 biological replicates. Error bars indicate standard deviation.
(TIF)

S3 Fig Changes of different lysolipids during the growth kinetic. Absolute amounts (μmol/g) are shown for monogalactosylmonoacylglycerol (MGMG), digalactosylmonoacylglycerol (DGMG), lysophosphatidylglycerol (LPG), monoacylglyceroltrimethyhomoserine (MGTS) lysophosphatidylcholine (LPC), lysophosphatidyethanolamine (LPE) and lysophosphatidylserine (LPS). For lysophosphatidylinositol (LPI) and lysophosphatidic acid (LPA) the relative amounts are depicted. Samples were taken at 0d, 2d, 5d and 7d for replete conditions (blue, tones are getting darker with increasing days). Sampling for N-deplete conditions were done at 0d, 2d, 3d and 6d. Normal light is shown in red (tones are getting darker with increasing days) and high light in green (tones are getting darker with increasing days). For sulfoquinovosylmonoacylglycerol (SQMG) no lipids were detectable. Data are mean values of 3 biological replicates. Error bars indicate standard deviation.
(TIF)

S4 Fig Comparison of the total amount of the different lipid classes under replete and N-deplete conditions. The total lipid content of the major lipid classes of the last time point of growth kinetic was calculated from the values shown in Figs 2 and 3. Triacylglycerol (TAG), diacylglycerol (DAG), monogalactosyldiacylglycerol (MGDG), digalactosyldiacylglycerols (DGDG), sulfoquinovosyldiacylglycerol (SQDG), phosphatidylglycerol (PG), diacylglyceroltrimethyhomoserine (DGTS), phosphatidylcholine (PC), phosphatidylethanolamine (PE), phosphatidylserine (PS). Phosphatidylinositol (PI) and phosphatidic acid (PA) are not displayed because these lipids have not been quantified. Data are mean values of 3 biological replicates. Error bars indicate standard deviation.
(TIF)

S5 Fig Principle component analysis (PCA) of the metabolome of *Phaeodactylum tricornutum* under replete and N-deplete conditions. *Phaeodactylum tricornutum* cultures grown under replete condition and normal light (NL), or grown under N-deplete conditions (-N) and normal light (NL) or high light (HL) were harvested at the indicated time points and extracted by two phase partitioning. The fingerprint of metabolites of the polar extraction phase was generated by UPLC-TOF-MS analysis. PCA analysis was performed by the software tool MarVis (MarkerVisualization, http://marvis.gobics.de). Data represent 3 biological replicates for each treatment.
(TIF)

S6 Fig High resolution MS/MS analyses by UHPLC-ESI-QTOF-MS. Shown are fragmentation patterns at 10–15 eV collision energies in positive (DMSP, dimethylsulfoniobutyrate and O-propanoyl-carnitin) or negative (methylsulfate) ionization mode, as well as the corresponding chemical formula for each compound based on the fragmentation pattern.
(TIF)

S1 Table Raw data table of the lipid analysis. In this table, the raw data of the different lipid molecular species are listed. The table is divided in absolute amounts [μmol/g] and relative values [%].
(XLSX)

S2 Table Main molecular species of each lipid class under different conditions. In this table, the major molecular species for each lipid class and condition are given as relative value.
(TIF)

S3 Table Raw data table–Metabolite fingerprinting. (XLSX)

S4 Table Raw data table of metabolites. Trimethylsilyl (TMS) and methoxyamine (MEOX) derivatives of the metabolites were analysed by GC-MS. Samples were measured in two (timepoint 0 d) or three biological replicates (all other samples). The standard deviation (SD) is given. Metabolites were identified either by their retention time and spectra compared with an external standard run on the same instrument (ES) or by their spectrum alone based on a spectrum library (SL) [Kopka J, Schauer N, Krueger S, Birkemeyer C, Usadel B, Bergmuller E, et al. GMD@CSB.DB: the Golm Metabolome Database. Bioinformatics. 2005;21: 1635–8]. The data is normalized with the average of all samples set to 1.
(XLSX)

S5 Table Raw data table of analytes. Trimethylsilyl (TMS) and methoxyamine (MEOX) derivatives of the metabolites were analysed by GC-MS. Samples were measured in two (timepoint 0 d) or three biological replicates (all other samples). Values were calculated from the integration results using the quantification mass to charge ratio (quant *m*/*z*) at the retention time (RT) depicted in the analyte sheets. The retention index (RI) was calculated based on an alkane standard. Values were divided by the value of the internal standard (Ribitol 5TMS) and the dry weight. For ribitol, the integral of the given *m*/*z* is depicted. In the case the metabolites were represented by more than one analyte the values were added based on the total ion count.
(XLSX)

## Abbreviations

Chl*a*: chlorophyll *a*
DAG: diacylglycerol
DGAT: acyl-CoA:diacylglycerol acyltransferase
DGDG: digalactosyldiacylglycerol
DGTA: diacylglycerylhydroxymethyltrimethyl-β-alanine
DGTS: diacylglyceroltrimethyhomoserine
DI-nanoESI-MS/MS: direct infusion-nano electrospray ionization-mass spectrometry
GL: glycolipids
MGDG: monogalactosyldiacylglycerol
NL: non-polar or neutral lipids
PA: phosphatidic acid
PC: phosphatidylcholine
PDAT: phospholipid: diacylglycerol acyltransferase
PE: phosphatidyethanolamine
PG: phosphatidylglycerol
PI: phosphatidylinositol
PL: phospholipids
PS: phosphatidylserine
SQDG: sulfoquinovosyldiacylglycerol
TAG: triacylglycerol

## References

[1] Borowitzka MA, Moheimani NR. Sustainable biofuels from algae. Mitigation and Adaptation Strategies for Global Change. 2013;18: 13–25.

[2] Carlsson AS, Yilmaz JL, Green AG, Stymne S, Hofvander P. Replacing fossil oil with fresh oil–with what and for what? Eur J Lipid Sci and Technol. 2011;113: 812–31. 22102794 10.1002/ejlt.201100032 PMC3210827

[3] Li-Beisson Y, Peltier G. Third-generation biofuels: current and future research on microalgal lipid biotechnology. OCL. 2013;20: D606.

[4] Chisti Y. Biodiesel from microalgae. Biotechnology Advances. 2007;25: 294–306. 10.1016/j.biotechadv.2007.02.001 17350212

[5] Hildebrand M, Davis AK, Smith SR, Traller JC, Abbriano R. The place of diatoms in the biofuels industry. Biofuels. 2012;3: 221–40.

[6] d'Ippolito G, Sardo A, Paris D, Vella F, Adelfi M, Botte P, et al Potential of lipid metabolism in marine diatoms for biofuel production. Biotechnol Biofuels. 2015;8: 28 10.1186/s13068-015-0212-4 25763104 PMC4355990

[7] Levitan O, Dinamarca J, Hochman G, Falkowski PG. Diatoms: a fossil fuel of the future. Trends Biotechnol. 2014;32: 117–24. 10.1016/j.tibtech.2014.01.004 24529448

[8] De Martino A, Meichenin A, Shi J, Pan K, Bowler C. Genetic and phenotypic characterization of *Phaeodactylum tricornutum* (Bacillariophyceae) accessions. J Phycol. 2007;43: 992–1009.

[9] Abida H, Dolch L-J, Mei C, Villanova V, Conte M, Block MA, et al Membrane glycerolipid remodeling triggered by nitrogen and phosphorus starvation in *Phaeodactylum tricornutum*. Plant Physiol. 2015;167: 118–36. 10.1104/pp.114.252395 25489020 PMC4281014

[10] Simionato D, Block MA, La Rocca N, Jouhet J, Marechal E, Finazzi G, et al The response of *Nannochloropsis gaditana* to nitrogen starvation includes *de novo* biosynthesis of triacylglycerols, a decrease of chloroplast galactolipids, and reorganization of the photosynthetic apparatus. Eukaryot Cell. 2013;12: 665–76. 10.1128/EC.00363-12 23457191 PMC3647774

[11] Allen JW, DiRusso CC, Black PN. Triacylglycerol synthesis during nitrogen stress involves the prokaryotic lipid synthesis pathway and acyl chain remodeling in the microalgae *Coccomyxa subellipsoidea*. Algal Res. 2015;10: 110–20.

[12] Rezanka T, Nedbalová L, Procházková L, Sigler K. Lipidomic profiling of snow algae by ESI-MS and silver-LC/APCI-MS. Phytochemistry. 2014;100: 34–42. 10.1016/j.phytochem.2014.01.017 24548555

[13] Kim S-H, Ahn HM, Lim SR, Hong S-J, Cho B-K, Lee H, et al Comparative lipidomic profiling of two *Dunaliella tertiolecta* strains with different growth temperatures under nitrate-deficient conditions. J Agric Food Chem. 2015;63: 880–7. 10.1021/jf502967k 25549757

[14] Bromke MA, Giavalisco P, Willmitzer L, Hesse H. Metabolic analysis of adaptation to short-term changes in culture conditions of the marine diatom *Thalassiosira pseudonana*. PLoS One. 2013;8: e67340 10.1371/journal.pone.0067340 23799147 PMC3682967

[15] Liu B, Benning C. Lipid metabolism in microalgae distinguishes itself. Curr Op Biotechnol. 2013;24: 300–9. 10.1016/j.copbio.2012.08.008 22981869

[16] Falkowski PG, Katz ME, Knoll AH, Quigg A, Raven JA, Schofield O, et al The evolution of modern eukaryotic phytoplankton. Science. 2004;305: 354–60. 10.1126/science.1095964 15256663

[17] Hockin NL, Mock T, Mulholland F, Kopriva S, Malin G. The response of diatom central carbon metabolism to nitrogen starvation is different from that of green algae and higher plants. Plant Physiol. 2012;158: 299–312. 10.1104/pp.111.184333 22065419 PMC3252072

[18] Guschina IA, Harwood JL. Algal lipids and their metabolism Algae for Biofuels and Energy: Springer; 2013 p. 17–36.

[19] Cañavate JP, Armada I, Ríos JL, Hachero-Cruzado I. Exploring occurrence and molecular diversity of betaine lipids across taxonomy of marine microalgae. Phytochemistry. 2016;124: 68–78. 10.1016/j.phytochem.2016.02.007 26895707

[20] Li S, Xu J, Chen J, Chen J, Zhou C, Yan X. The major lipid changes of some important diet microalgae during the entire growth phase. Aquaculture. 2014;428: 104–10.

[21] Dembitsky VM. Betaine ether-linked glycerolipids: Chemistry and biology. Prog Lipid Res. 1996;35: 1–51. 9039425 10.1016/0163-7827(95)00009-7

[22] Riekhof WR, Sears BB, Benning C. Annotation of genes involved in glycerolipid biosynthesis in *Chlamydomonas reinhardtii*: Discovery of the betaine lipid synthase BTA1Cr. Eukaryot Cell. 2005;4: 242–52. 10.1128/EC.4.2.242-252.2005 15701786 PMC549322

[23] Guschina IA, Harwood JL. Algal lipids and effect of the environment on their biochemistry Lipids in Aquatic Ecosystems: Springer; 2009 p. 1–24.

[24] Yang Z-K, Ma Y-H, Zheng J-W, Yang W-D, Liu J-S, Li H-Y. Proteomics to reveal metabolic network shifts towards lipid accumulation following nitrogen deprivation in the diatom *Phaeodactylum tricornutum*. J Appl Phycol. 2014;26: 73–82. 10.1007/s10811-013-0050-3 24600163 PMC3918386

[25] Bromke MA, Hochmuth A, Tohge T, Fernie AR, Giavalisco P, Burgos A, et al Liquid chromatography high-resolution mass spectrometry for fatty acid profiling. Plant J. 2015;81: 529–36. 10.1111/tpj.12739 25440443

[26] Xue J, Niu Y-F, Huang T, Yang W-D, Liu J-S, Li H-Y. Genetic improvement of the microalga *Phaeodactylum tricornutum* for boosting neutral lipid accumulation. Metab Eng. 2015;27: 1–9. 10.1016/j.ymben.2014.10.002 25447640

[27] Guihéneuf F, Leu S, Zarka A, Khozin-Goldberg I, Khalilov I, Boussiba S. Cloning and molecular characterization of a novel acyl-CoA:diacylglycerol acyltransferase 1-like gene (PtDGAT1) from the diatom *Phaeodactylum tricornutum*. FEBS J. 2011;278: 3651–66. 10.1111/j.1742-4658.2011.08284.x 21812932

[28] Solovchenko A, Khozin-Goldberg I, Cohen Z, Merzlyak M. Carotenoid-to-chlorophyll ratio as a proxy for assay of total fatty acids and arachidonic acid content in the green microalga *Parietochloris incisa*. J Appl Phycol. 2009;21: 361–6.

[29] Matyash V, Liebisch G, Kurzchalia TV, Shevchenko A, Schwudke D. Lipid extraction by methyl-tert-butyl ether for high-throughput lipidomics. J Lipid Res. 2008;49: 1137–46. 10.1194/jlr.D700041-JLR200 18281723 PMC2311442

[30] Reich M, Göbel C, Kohler A, Buée M, Martin F, Feussner I, et al Fatty acid metabolism in the ectomycorrhizal fungus *Laccaria bicolor*. New Phytol. 2009;182: 950–64. 10.1111/j.1469-8137.2009.02819.x 19383096

[31] Hornung E, Pernstich C, Feussner I. Formation of conjugated Δ^11^Δ^13^-double bonds by Δ^12^-linoleic acid (1,4)-acyl-lipid-desaturase in pomegranate seeds. Eur J Biochem. 2002;269: 4852–9. 12354116 10.1046/j.1432-1033.2002.03184.x

[32] Bellaire A, Ischebeck T, Staedler Y, Weinhaeuser I, Mair A, Parameswaran S, et al Metabolism and development–integration of micro computed tomography data and metabolite profiling reveals metabolic reprogramming from floral initiation to silique development. New Phytol. 2014;202: 322–35. 10.1111/nph.12631 24350948 PMC4283998

[33] Larson TR, Graham IA. Technical Advance: a novel technique for the sensitive quantification of acyl CoA esters from plant tissues. Plant J. 2001;25: 115–25. 11169187 10.1046/j.1365-313x.2001.00929.x

[34] Haynes CA, Allegood JC, Sims K, Wang EW, Sullards MC, Merrill AH, Jr. Quantitation of fatty acyl-coenzyme As in mammalian cells by liquid chromatography-electrospray ionization tandem mass spectrometry. J Lipid Res. 2008;49: 1113–25. 10.1194/jlr.D800001-JLR200 18287618 PMC2311451

[35] König S, Feussner K, Kaever A, Landesfeind M, Thurow C, Karlovsky P, et al Soluble phenylpropanoids are involved in the defense response of Arabidopsis against *Verticillium longisporum*. New Phytol. 2014;202: 823–37. 10.1111/nph.12709 24483326

[36] Kaever A, Landesfeind M, Feussner K, Mosblech A, Heilmann I, Morgenstern B, et al MarVis-Pathway: integrative and exploratory pathway analysis of non-targeted metabolomics data. Metabolomics. 2015;11: 764–77. 10.1007/s11306-014-0734-y 25972773 PMC4419191

[37] Kaever A, Lingner T, Feussner K, Göbel C, Feussner I, Meinicke P. MarVis: a tool for clustering and visualization of metabolic biomarkers. BMC Bioinform. 2009;10: 92. 10.1186/1471-2105-10-92 PMC2666665 19302701

[38] Alipanah L, Rohloff J, Winge P, Bones AM, Brembu T. Whole-cell response to nitrogen deprivation in the diatom *Phaeodactylum tricornutum*. J Exp Bot. 2015;66: 6281–96. 10.1093/jxb/erv340 26163699 PMC4588885

[39] Rohn H, Junker A, Hartmann A, Grafahrend-Belau E, Treutler H, Klapperstuck M, et al VANTED v2: a framework for systems biology applications. BMC Syst Biol. 2012;6: 139 10.1186/1752-0509-6-139 23140568 PMC3610154

[40] Lenky CC, McEntyre CJ, Lever M. Measurement of marine osmolytes in mammalian serum by liquid chromatography–tandem mass spectrometry. Anal Biochem. 2012;420: 7–12. 10.1016/j.ab.2011.09.013 21982861

[41] Bucciarelli E, Sunda WG. Influence of CO_2_, nitrate, phosphate, and silicate limitation on intracellular dimethylsulfoniopropionate in batch cultures of the coastal diatom *Thalassiosira pseudonana*. Limnol Oceanogr. 2003;48: 2256–65.

[42] Rashed MS, Bucknall MP, Little D, Awad A, Jacob M, Alamoudi M, et al Screening blood spots for inborn errors of metabolism by electrospray tandem mass spectrometry with a microplate batch process and a computer algorithm for automated flagging of abnormal profiles. Clin Chem. 1997;43: 1129–41. 9216448

[43] Breuer G, Lamers PP, Martens DE, Draaisma RB, Wijffels RH. The impact of nitrogen starvation on the dynamics of triacylglycerol accumulation in nine microalgae strains. Biores Technol. 2012;124: 217–26. 10.1016/j.biortech.2012.08.003 22995162

[44] Bromke MA, Sabir JS, Alfassi FA, Hajarah NH, Kabli SA, Al-Malki AL, et al Metabolomic profiling of 13 diatom cultures and their adaptation to nitrate-limited growth conditions. PLoS ONE. 2015;10: e0138965 10.1371/journal.pone.0138965 26440112 PMC4595471

[45] KaiXian Q, Borowitzka MA. Light and nitrogen deficiency effects on the growth and composition of *Phaeodactylum tricornutum*. Appl Biochem Biotechnol. 1993;38: 93–103.

[46] James GO, Hocart CH, Hillier W, Chen H, Kordbacheh F, Price GD, et al Fatty acid profiling of *Chlamydomonas reinhardtii* under nitrogen deprivation. Biores Technol. 2011;102: 3343–51. 10.1016/j.biortech.2010.11.051 21146403

[47] Zhekisheva M, Boussiba S, Khozin‐Goldberg I, Zarka A, Cohen Z. Accumulation of oleic acid in *Haematococcus pluvialis* (Chlorophyceae) under nitrogen starvation or high light is correlated with that of astaxanthin esters. J Phycol. 2002;38: 325–31.

[48] Solovchenko AE, Merzlyak MN, Chivkunova OB, Reshetnikova IV, Khozina-Goldberg I, Didi-Cohen S, et al Effects of illumination and nitrogen starvation on accumulation of arachidonic acid by the microalga *Parietochloris incisa*. Moscow Univ Biol Sci Bull. 2008;63: 44–8.

[49] Giroud C, Gerber A, Eichenberger W. Lipids of *Chlamydomonas reinhardtii*. Analysis of molecular species and intracellular site(s) of biosynthesis. Plant Cell Physiol. 1988;29: 587–95.

[50] Armada I, Hachero-Cruzado I, Mazuelos N, Ríos JL, Manchado M, Canavate JP. Differences in betaine lipids and fatty acids between *Pseudoisochrysis paradoxa* VLP and *Diacronema vlkianum* VLP isolates (Haptophyta). Phytochemistry. 2013;95: 224–33. 10.1016/j.phytochem.2013.07.024 23954077

[51] Van Mooy BAS, Fredricks HF, Pedler BE, Dyhrman ST, Karl DM, Koblizek M, et al Phytoplankton in the ocean use non-phosphorus lipids in response to phosphorus scarcity. Nature. 2009;458: 69–72. 10.1038/nature07659 19182781

[52] Zienkiewicz K, Du Z-Y, Ma W, Vollheyde K, Benning C. Stress-induced neutral lipid biosynthesis in microalgae—Molecular, cellular and physiological insights. Biochim Biophys Acta. 2016;1861: 1269–1281. 10.1016/j.bbalip.2016.02.008 26883557

[53] Danielewicz MA, Anderson LA, Franz AK. Triacylglycerol profiling of marine microalgae by mass spectrometry. J Lipid Res. 2011;52: 2101–8. 10.1194/jlr.D018408 21840867 PMC3196241

[54] Gong Y, Zhang J, Guo X, Wan X, Liang Z, Hu CJ, et al Identification and characterization of PtDGAT2B, an acyltransferase of the DGAT2 acyl-Coenzyme A: Diacylglycerol acyltransferase family in the diatom *Phaeodactylum tricornutum*. FEBS Lett. 2013;587: 481–7. 10.1016/j.febslet.2013.01.015 23337871

[55] Hamilton ML, Haslam RP, Napier JA, Sayanova O. Metabolic engineering of Phaeodactylum tricornutum for the enhanced accumulation of omega-3 long chain polyunsaturated fatty acids. Metab Eng. 2014;22: 3–9. 10.1016/j.ymben.2013.12.003 24333273 PMC3985434

[56] Légeret B, Schulz-Raffelt M, Nguyen HM, Auroy P, Beisson F, Peltier G, et al Lipidomic and transcriptomic analyses of *Chlamydomonas reinhardtii* under heat stress unveil a direct route for the conversion of membrane lipids into storage lipids. Plant, Cell Environm. 2016;39: 834–47. 10.1111/pce.12656 26477535

[57] Allen AE, Dupont CL, Obornik M, Horak A, Nunes-Nesi A, McCrow JP, et al Evolution and metabolic significance of the urea cycle in photosynthetic diatoms. Nature. 2011;473: 203–7. 10.1038/nature10074 21562560

[58] Kim J, Fabris M, Baart G, Kim MK, Goossens A, Vyverman W, et al Flux balance analysis of primary metabolism in the diatom *Phaeodactylum tricornutum*. Plant J. 2016;85: 161–76. 10.1111/tpj.13081 26590126

[59] Ceusters J, Godts C, Peshev D, Vergauwen R, Dyubankova N, Lescrinier E, et al Sedoheptulose accumulation under CO2 enrichment in leaves of *Kalanchoe pinnata*: a novel mechanism to enhance C and P homeostasis? J Exp Bot. 2013;64: 1497–507. 10.1093/jxb/ert010 23378377 PMC3617823

[60] Gebser B, Pohnert G. Synchronized regulation of different zwitterionic metabolites in the osmoadaption of phytoplankton. Mar Drugs. 2013;11: 2168–82. 10.3390/md11062168 23774888 PMC3721227

