# Supplementary figures and images for: Metabolome Analysis Reveals Betaine Lipids as Major Source for Triglyceride Formation, and the Accumulation of Sedoheptulose during Nitrogen-Starvation of Phaeodactylum tricornutum
Source: PLoS One. 2016 Oct 13;11(10):e0164673. doi: 10.1371/journal.pone.0164673 (PMC5063337; doi:10.1371/journal.pone.0164673)

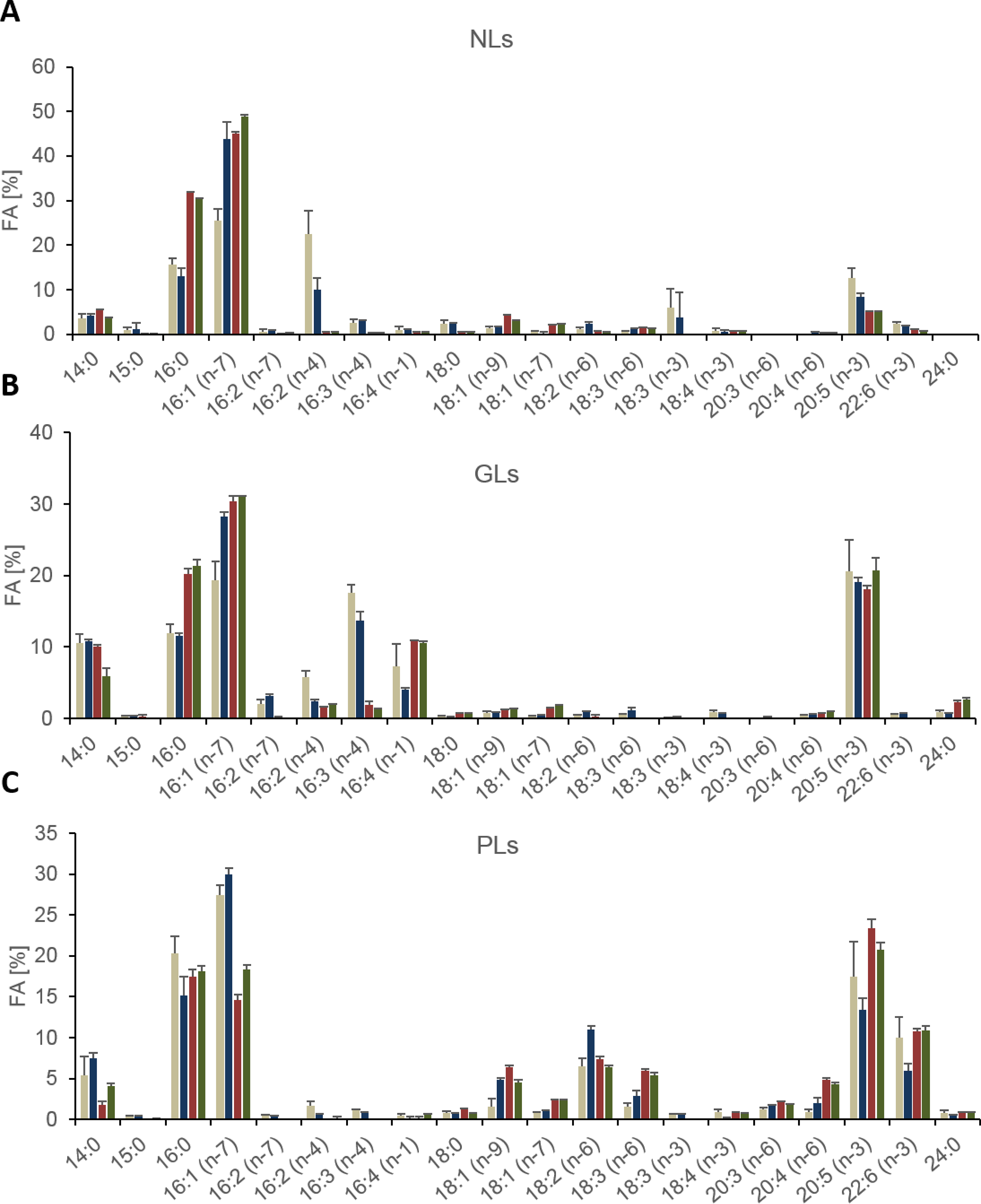

Supplement: S1 Fig — FA profile of neutral lipids (A), glycolipids (B) and phospholipids (C). The diagrams shows day 0 and the last time point of each condition. Day 0 comprises the mean of all conditions (beige). Day 7 of replete conditions is shown in blue, day 6 of N-deplete with normal light in red and day 6 of N-deplete with high light in green. Data are mean values of 3 biological replicates, for day 0 9 biological replicates were used. Error bars indicate standard deviation. (TIF) [file pone.0164673.s001.tif]

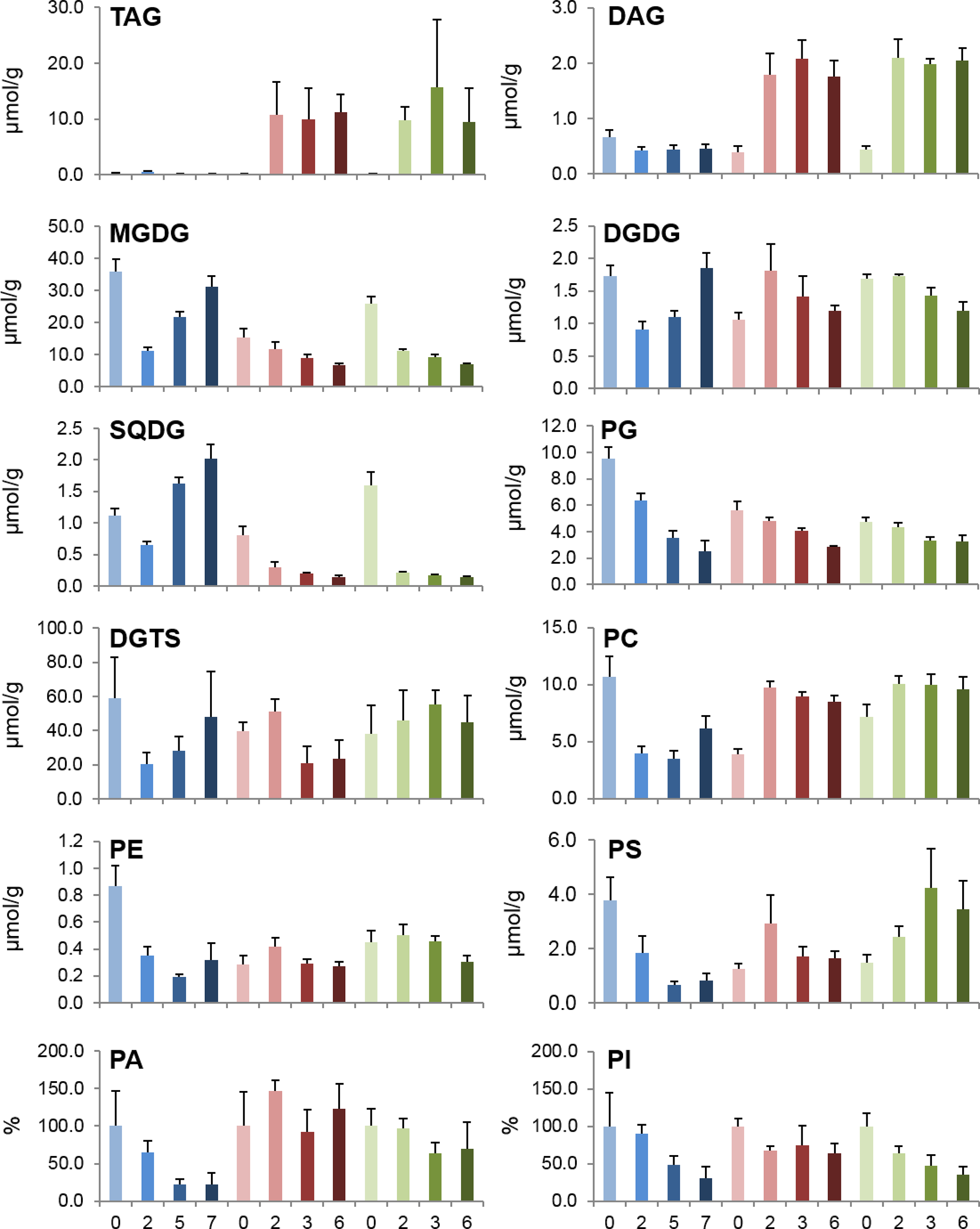

Supplement: S2 Fig — Absolute amounts (μmol/g) are given for triaclyglycerol (TAG), diacylglycerol (DAG), monogalactosyldiacylglycerol (MGDG), digalactosyldiacylglycerol (DGDG), sulfoquinovosyldiacylglycerol (SQDG), phosphatidylglycerol (PG), diacylglyceroltrimethyhomoserine (DGTS), phosphatidylcholine (PC), phosphatidyethanolamine (PE), and phosphatidylserine (PS). For phosphatidylinositol (PI) and phosphatidic acid (PA) the relative amounts are depicted. Samples were taken at 0d, 2d, 5d and 7d for N-replete conditions (blue, tones are getting darker with increasing days). Sampling for N-deplete conditions were done at 0d, 2d, 3d and 6d. Normal light is shown in red (tones are getting darker with increasing days) and high light in green (tones are getting darker with increasing days). Data are mean values of 3 biological replicates. Error bars indicate standard deviation. (TIF) [file pone.0164673.s002.tif]

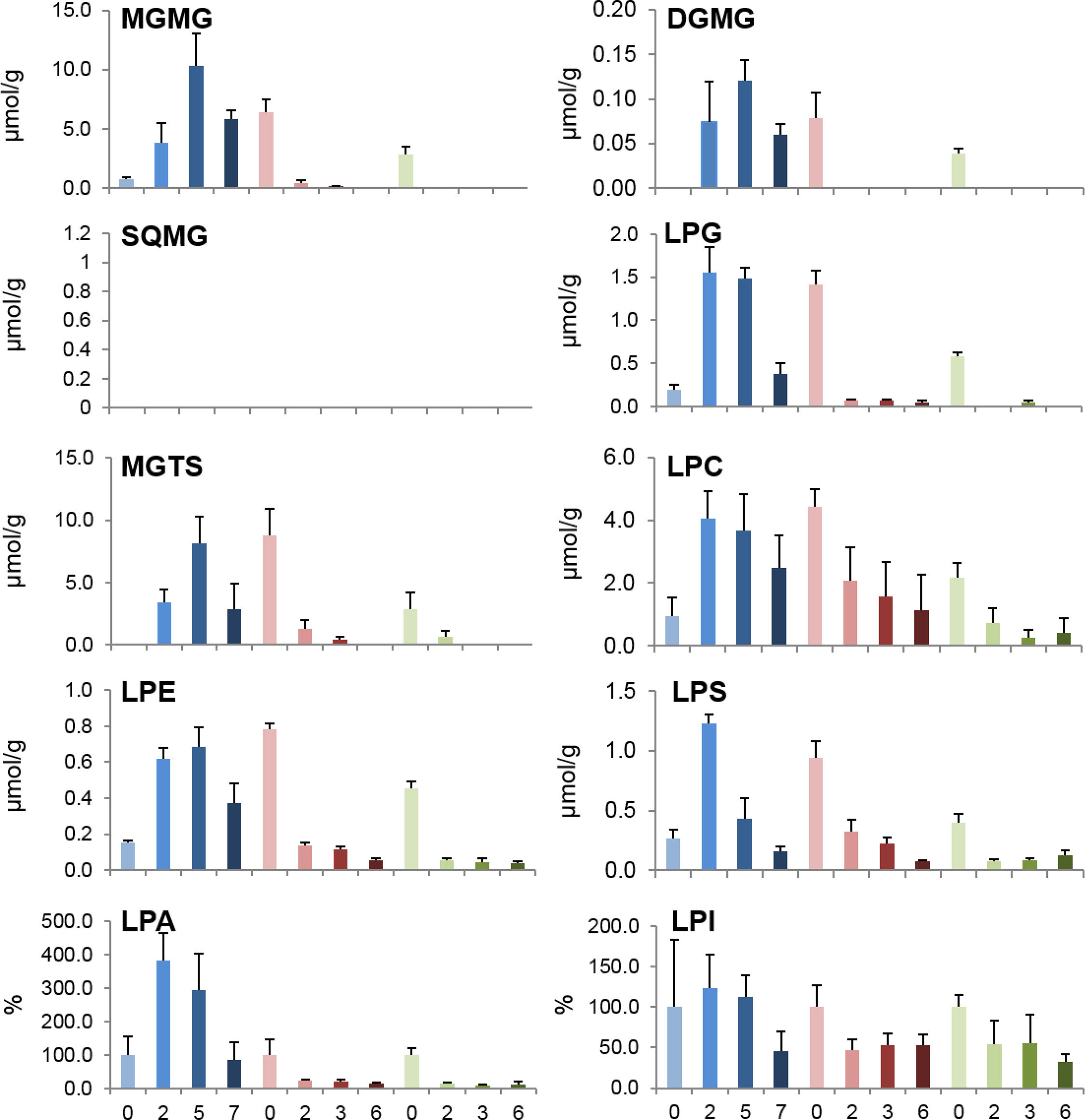

Supplement: S3 Fig — Absolute amounts (μmol/g) are shown for monogalactosylmonoacylglycerol (MGMG), digalactosylmonoacylglycerol (DGMG), lysophosphatidylglycerol (LPG), monoacylglyceroltrimethyhomoserine (MGTS) lysophosphatidylcholine (LPC), lysophosphatidyethanolamine (LPE) and lysophosphatidylserine (LPS). For lysophosphatidylinositol (LPI) and lysophosphatidic acid (LPA) the relative amounts are depicted. Samples were taken at 0d, 2d, 5d and 7d for replete conditions (blue, tones are getting darker with increasing days). Sampling for N-deplete conditions were done at 0d, 2d, 3d and 6d. Normal light is shown in red (tones are getting darker with increasing days) and high light in green (tones are getting darker with increasing days). For sulfoquinovosylmonoacylglycerol (SQMG) no lipids were detectable. Data are mean values of 3 biological replicates. Error bars indicate standard deviation. (TIF) [file pone.0164673.s003.tif]

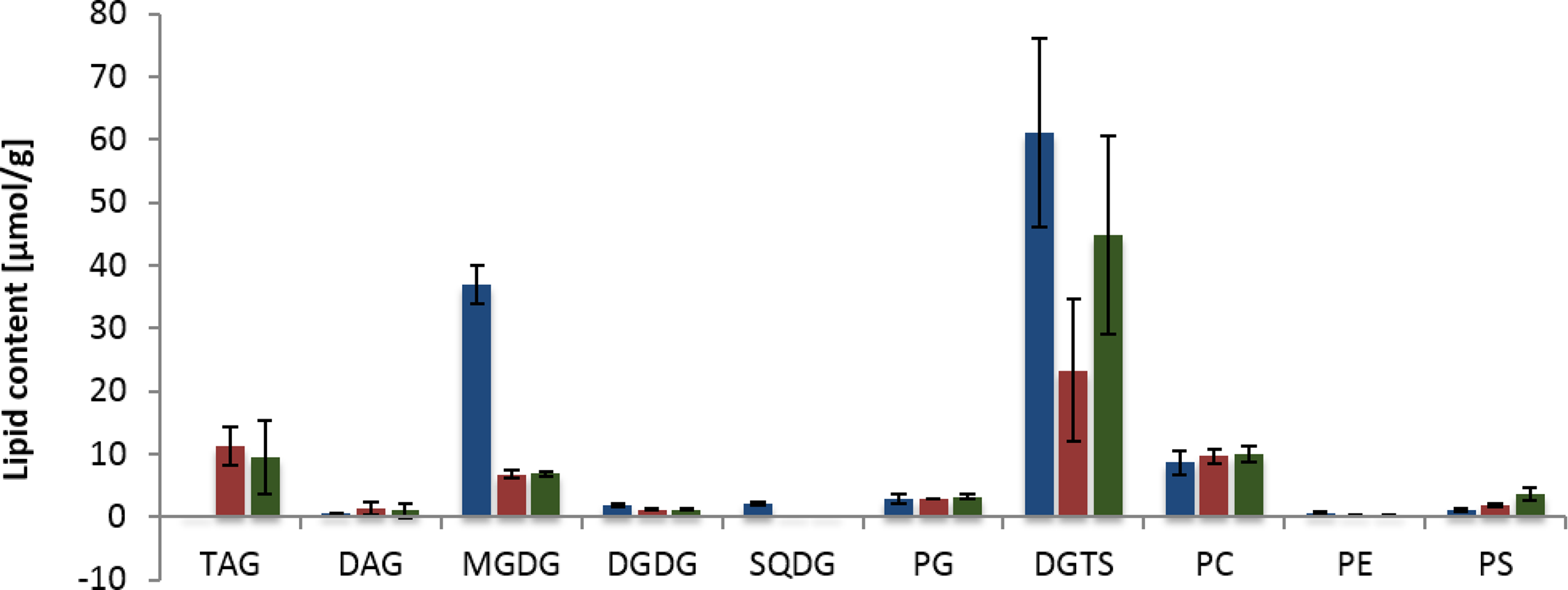

Supplement: S4 Fig — The total lipid content of the major lipid classes of the last time point of growth kinetic was calculated from the values shown in Figs 2 and 3. Triacylglycerol (TAG), diacylglycerol (DAG), monogalactosyldiacylglycerol (MGDG), digalactosyldiacylglycerols (DGDG), sulfoquinovosyldiacylglycerol (SQDG), phosphatidylglycerol (PG), diacylglyceroltrimethyhomoserine (DGTS), phosphatidylcholine (PC), phosphatidylethanolamine (PE), phosphatidylserine (PS). Phosphatidylinositol (PI) and phosphatidic acid (PA) are not displayed because these lipids have not been quantified. Data are mean values of 3 biological replicates. Error bars indicate standard deviation. (TIF) [file pone.0164673.s004.tif]

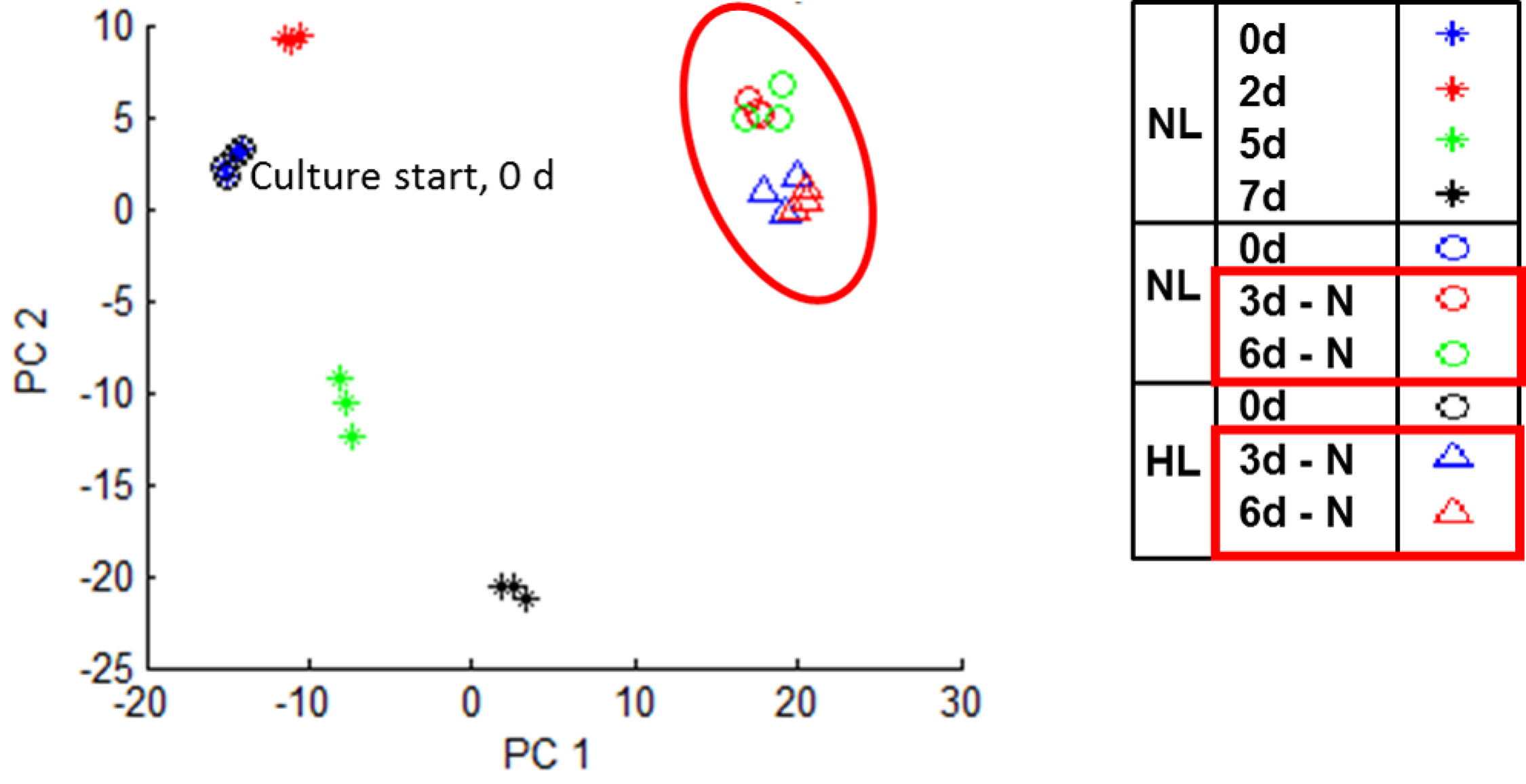

Supplement: S5 Fig — Phaeodactylum tricornutum cultures grown under replete condition and normal light (NL), or grown under N-deplete conditions (-N) and normal light (NL) or high light (HL) were harvested at the indicated time points and extracted by two phase partitioning. The fingerprint of metabolites of the polar extraction phase was generated by UPLC-TOF-MS analysis. PCA analysis was performed by the software tool MarVis (MarkerVisualization, http://marvis.gobics.de). Data represent 3 biological replicates for each treatment. (TIF) [file pone.0164673.s005.tif]

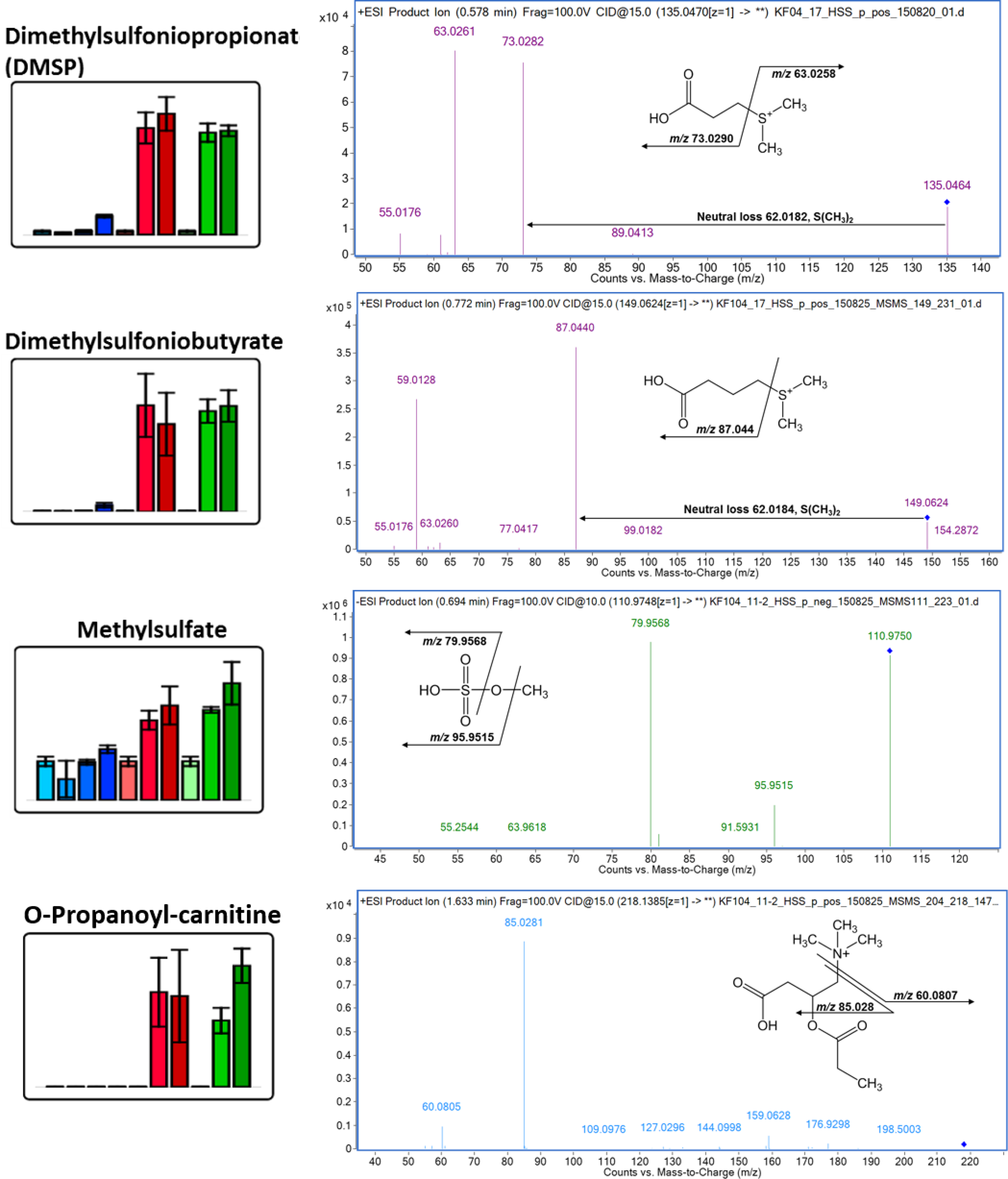

Supplement: S6 Fig — Shown are fragmentation patterns at 10–15 eV collision energies in positive (DMSP, dimethylsulfoniobutyrate and O-propanoyl-carnitin) or negative (methylsulfate) ionization mode, as well as the corresponding chemical formula for each compound based on the fragmentation pattern. (TIF) [file pone.0164673.s006.tif]

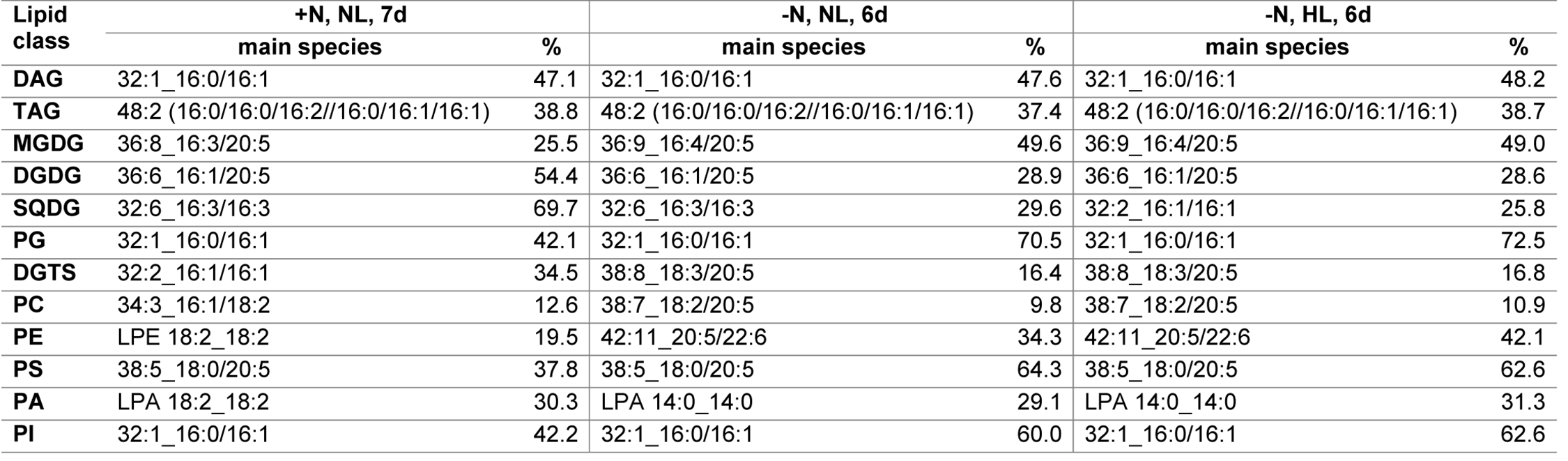

Supplement: S2 Table — In this table, the major molecular species for each lipid class and condition are given as relative value. (TIF) [file pone.0164673.s008.tif]
